# Sedentary behavior and physical activity are associated with risk of depression among adult and older populations: a systematic review and dose–response meta-analysis

**DOI:** 10.3389/fpsyg.2025.1542340

**Published:** 2025-03-17

**Authors:** Dawei Wang, Yuheng Zhang, Zhiguang Guo, Songtao Lu

**Affiliations:** ^1^School of Physical Education, Central China Normal University, Wuhan, China; ^2^College of Physical Education, Hubei Minzu University, Enshi, China; ^3^School of Sports, Wuhan University of Science and Technology, Wuhan, China; ^4^School of Sports Health, Hubei University of Chinese Medicine, Wuhan, China

**Keywords:** sedentary behavior, physical activity, accelerometry, depression, adults, older populations

## Abstract

**Background:**

Depression symptoms are commonly experienced by adults and older people; however, there is uncertainty concerning the associations of lifestyle with the risk of depression. This study systematically reviewed and meta-analyzed observational data to assess the link between instrumented sedentary behavior (i-SB) and physical activity (i-PA) measures and depression risk among adult and older populations.

**Methods:**

A systematic review across four databases was performed up to July 27, 2024, targeting studies linking i-SB, i-PA, and depression. The review included a dose–response meta-analysis, presenting results as odds ratios (OR) and 95% confidence intervals (95% CI).

**Results:**

Fifty-one studies, encompassing 1,318,687 participants, fulfilled the inclusion criteria. The comparison between the most and least sedentary groups yielded a pooled OR of 1.09 (95% CI 1.05–1.13). The comparison between the least and most active participant groups yielded pooled ORs of 0.96 (95% CI 0.93–0.98) for light activity (LPA), 0.91 (95% CI 0.86–0.96) for moderate-to-vigorous activity (MVPA), 0.93 (95% CI 0.90–0.96) for total physical activity (TPA), and 0.87 (95% CI 0.81–0.94) for steps per day. After adjusting i-PA, a lower OR for i-SB did not indicate a significant link to increased depression risk. Meta-regression analyses confirmed a dose–response relationship between SB, MVPA, daily steps, and depression.

**Conclusion:**

The association between i-SB and the risk of depression was not consistent with the results of previous self-reported studies. MVPA linked to the risk of depression was independent of i-SB, whereas the link between i-SB and the risk of depression was not independent of i-PA.

**Systematic review registration:**

https://www.crd.york.ac.uk/PROSPERO/display_record.php?RecordID=546666, identifier CRD42024546666.

## 1 Introduction

Mental health encompasses cognitive rationality, emotional equilibrium, behavioral suitability, interpersonal harmony, and adaptive flexibility, marking a state of well-being throughout an individual’s developmental trajectory. According to a 2017 survey conducted by the World Health Organization, nearly one billion individuals worldwide, a staggering figure, are grappling with various mental health challenges (World Health Organization, 2017). The 2020 WHO Guidelines highlight that regular physical activity in adults aged 18–64 yields substantial health benefits, including mental health improvements such as reduced anxiety and depression symptoms, along with enhanced cognitive function and better sleep quality, all of which collectively contribute to an individual’s overall well-being (World Health Organization, 2020). Physical activity (PA), defined as any bodily movement resulting from skeletal muscle contractions that lead to energy expenditure above basal levels (≥1.5 METs[Metabolic Equivalent of Task]), is associated with a multitude of benefits including physical fitness, mental health enhancement, and cognitive improvements (Ainsworth et al., 2011). PA can serve as a complementary or alternative strategy for mental health and behavioral function interventions (Murray et al., 2023). Sedentary behavior (SB), characterized by activities undertaken in a seated, reclined, or supine position during periods of wakefulness, involves an energy expenditure equivalent to or less than 1.5 metabolic equivalents (METs) (Ainsworth et al., 2011). This includes various everyday activities, such as viewing television, engaging with computers, and maintaining a seated posture while utilizing transportation (Tremblay et al., 2017). These behaviors typify a low level of energy expenditure, in contrast to the higher energy demands of PA.

Empirical evidence, as documented in prior investigations, has unequivocally established a link between distinct intensities of PA (Rautio and Seppänen, 2024) and SB (Siwa et al., 2023) and the prevalence and incidence of depressive symptoms. Precisely speaking, extant studies have firmly corroborated the notion that the reallocation of a mere 15-min duration of moderate-to-vigorous PA (MVPA) to SB precipitates a marked elevation in the intensity of depressive symptoms, quantifiable by an increment of 0.46 units on a relevant measurement scale (Murray et al., 2023). Interestingly, the aggregate duration of sedentary time exhibits a significant relationship with escalated depressive symptoms exclusively within the male population (Wemeck et al., 2022). These findings collectively underscore the intricate interplay between sedentary habits, PA patterns, and mental health, particularly in the context of depressive symptom management. Second, previous research has recognized PA as a potentially effective means of preventing mental health issues (Denche-Zamorano et al., 2023). Previous research has confirmed that an increase in PA is associated with a reduction in the risk of depression (B: −0.043, 95% confidence intervals-95% CI: −0.071 to −0.016) (Iob et al., 2023). Furthermore, in adults over the age of 65, depressive symptoms have been associated with a reduction in the total duration of PA at an intensity greater than 3 METs (Dhakal et al., 2023). In summary, PA has a positive causal effect on the prevention of depression, whereas excessive sedentary time may increase the risk of anxiety and diminish mental health status.

However, determining the precise amount of PA required to substantially reduce the risk of depression and, conversely, identifying the threshold of SB that significantly elevates exposure to the risk of depression remains a question worthy of contemplation and further investigation. Previous studies have shown that moderate-to-vigorous PA (MVPA) is significantly associated with a reduced likelihood of depression (pooled odds ratios, OR: 0.817, 95% CI: 0.678 to 0.985). Replacing 30 min of SB with 30 min of MVPA was linked to a decrease in the odds of depression (OR: 0.815, 95% CI: 0.669 to 0.992). Conversely, substituting 30 min of MVPA with 30 min of SB was associated with an increased risk of depression (OR: 1.227, 95% CI: 1.008 to 1.495) (Park et al., 2024). This suggests that converting 30 min of sitting time into an equivalent duration of MVPA can lower the probability of developing depression, whereas replacing 30 min of MVPA with sitting can increase the risk of depression (Park et al., 2024). This indicates that to prevent depression in the elderly, it is advisable to accumulate a greater amount of MVPA throughout the day rather than simply engaging in light PA (LPA) (Chen et al., 2023). This indicates that allocating a greater proportion of one’s daily PA profile to MVPA as opposed to SB or LPA is associated with a notable decrease in the risk of developing depressive disorders (Blodgett et al., 2023). Consequently, the exact delineation of the association between SB and PA concerning depression, in conjunction with a thorough comparative examination of the effect sizes, assumes crucial significance in the conception of potent intervention models geared toward the amelioration of depressive conditions. This knowledge will be instrumental in guiding personalized lifestyle modifications and public health policies designed to optimize mental health outcomes.

Due to the limitations of questionnaires in accurately capturing unstructured PA and the tendency of adults to overestimate PA while underestimating SB (Ryan et al., 2018; Dyrstad et al., 2014), previous meta-analyses and observational studies may have been compromised in their accuracy of estimating the risk of depression due to these subjective factors. Furthermore, self-reported measures tend to overestimate PA and underestimate SB (Ryan et al., 2018). A previous study reported that a total of 119 (76%) young-to-middle-aged adults and 72 (90%) older adults reported that they met the recommended Dutch PA guidelines, while I-PA (instrumented measures) indicated that 37 (24%) and 13 (16%) participants, respectively, objectively met these criteria (Rojer et al., 2020). Given that objective measurements can provide more accurate data, our study exclusively used objective measures of PA and SB, which is a key strength. In other words, using objective instruments such as accelerometers and pedometers, more researchers have quantified the actual association among SB, PA, and depression, effectively mitigating the influence of participants’ subjective factors on the precision of depression risk estimation. These approaches maximally utilize all available data and generate more robust and reliable statistical results.

The aim of this systematic review and meta-analysis is to quantify the link between instrument-assessed sedentary behavior (i-SB) and physical activity (i-PA) with depression. It involves assessing the measurable impacts of i-SB and i-PA and outlining the dose–response relationship with depressive symptoms. Our hypothesis posited that escalated i-SB coupled with diminished i-PA correlates with an augmented risk of depression, which can potentially be ameliorated by increased i-PA or reduced i-SB. The insight derived from this investigation reinforces the pivotal function of PA in curtailing the likelihood of depression and sustaining mental health integrity, concurrently accentuating the importance of judiciously managing sedentary intervals. The empirical evidence garnered herein offers a robust scientific rationale for formulating public health initiatives aimed at endorsing PA and discouraging protracted sedentary periods with the overarching goal of enhancing mental health resilience within the population.

## 2 Methods

### 2.1 Search strategy

The systematic review protocol is registered with PROSPERO under CRD42024546666. Following PRISMA guidelines, a rigorous search of PubMed, Scopus, PsycINFO, and SPORTDiscus (via EBSCO) was conducted to identify literature on the link between physical activity and depression, from inception to July 27, 2024. We conducted a search for articles using specific search terms such as “depression OR depressive symptoms,” “physical activity OR exercise OR physical training,” “sedentary behavior OR behavior,” “sedentary OR sedentary behaviors,” “sedentary lifestyle OR lifestyle,” “sedentary OR physical sedentary OR physical inactivity,” “adult OR older,” and “accelerometry OR accelerometer OR pedometer OR monitoring.” We focused on prospective studies. Furthermore, we systematically reviewed the reference lists of all included studies to identify additional eligible articles, without language restrictions. The comprehensive search methodology is detailed in Supplementary Tables S1–S4.

### 2.2 Study selection

Two of three reviewers (DW and ZG) conducted a comprehensive assessment of titles, abstracts, and full texts to ascertain study eligibility per predefined criteria, with disagreements resolved by a third reviewer (SL). The literature management was facilitated by EndNote (Version X8.2, Clarivate Analytics, Philadelphia, PA, USA). Studies were eligible if they were published in English or Chinese and met the following criteria: (1) observational cohort, cross-sectional, case–control, or Mendelian randomization studies assessing sedentary behavior (SB) or physical activity (PA) via pedometry or accelerometry; (2) included depression, depressive symptoms, or major depression as outcomes; (3) involved adults aged 18 or older; (4) targeted adults generally, not a specific disease group; and (5) reported ORs, RRs, HRs, and 95% CIs for the association between PA/SB and depression risk, or provided data to calculate these metrics. In cases of multiple studies on the same population, only those with longer follow-ups or larger sample sizes were included. Researchers independently identified eligible studies and collectively determined their inclusion based on the established criteria.

### 2.3 Statistical analyses

This meta-analysis was performed utilizing STATA software (v16.0), with all statistical tests being two-tailed. The analysis involved a two-step approach for categorical and continuous variables to assess the link between physical activity (PA) and depression risk. The results are presented as odds ratios (ORs) with 95% confidence intervals (CIs). Studies were categorized as cohort, cross-sectional, or Mendelian randomization for pooled analysis. Random-effect models were applied to compute pooled ORs. Heterogeneity was assessed using the I2 statistic, with values of 25, 50, and 75% indicating low, moderate, and high heterogeneity, respectively (Higgins et al., 2019). The significance level for evaluating heterogeneity was established at 0.05. The tests conducted by Egger and Begg were employed to ascertain the presence of publication bias (Higgins et al., 2019). To perform sensitivity analysis, studies were sequentially omitted to evaluate their influence on the pooled effect of the remaining studies. Data were categorized into subgroups by study design, gender, age, research domain, study rigor, and control for confounders, followed by meta-analysis. Meta-regression was used to assess heterogeneity across studies.

To assess the relationship between dosage and response, we calculated weekly cumulative durations of physical activity (PA) and sedentary behavior (SB) for each effect value from the literature, assuming constant PA and SB durations over the follow-up period. The median was used to define the reference dose. Intervals below 0.5 were adjusted to 0.25, and when the upper open interval reached 1 or more, the difference between intermediate dose intervals was set at 0.25, resulting in an exposure value of 1.25 (Zhang et al., 2015).

The researchers utilized a robust error meta-regression technique, as outlined by Xu et al., to determine the slopes of the continuous dose–response relationship (nonlinear trends) and their corresponding 95% confidence intervals (Xu and Doi, 2018). This was done by analyzing the natural logarithms of the reported odds ratios (ORs) and confidence intervals (CIs) across several categories of physical activity (PA) and sedentary behavior (SB) measurements. This approach employs a single-stage method, treating each study as a sample cluster. It addresses within-study correlations using clustered robust standard errors. The Stata XBLC function was applied to derive a dose–response curve, based on the model’s goodness-of-fit assessment (Zhang et al., 2015).

### 2.4 Quality assessment

The Newcastle-Ottawa Scale (NOS) was utilized for quality assessment of the literature (Wells et al., 2000). Quality is directly proportional to the NOS score, with a maximum possible score of 9. Studies were categorized as low, moderate, or high quality based on NOS scores of 0–3, 4–6, and 7–9, respectively. Discrepancies in quality evaluation were resolved through group consensus to determine the final score.

## 3 Results

### 3.1 Description of included studies

**Figure 1:**
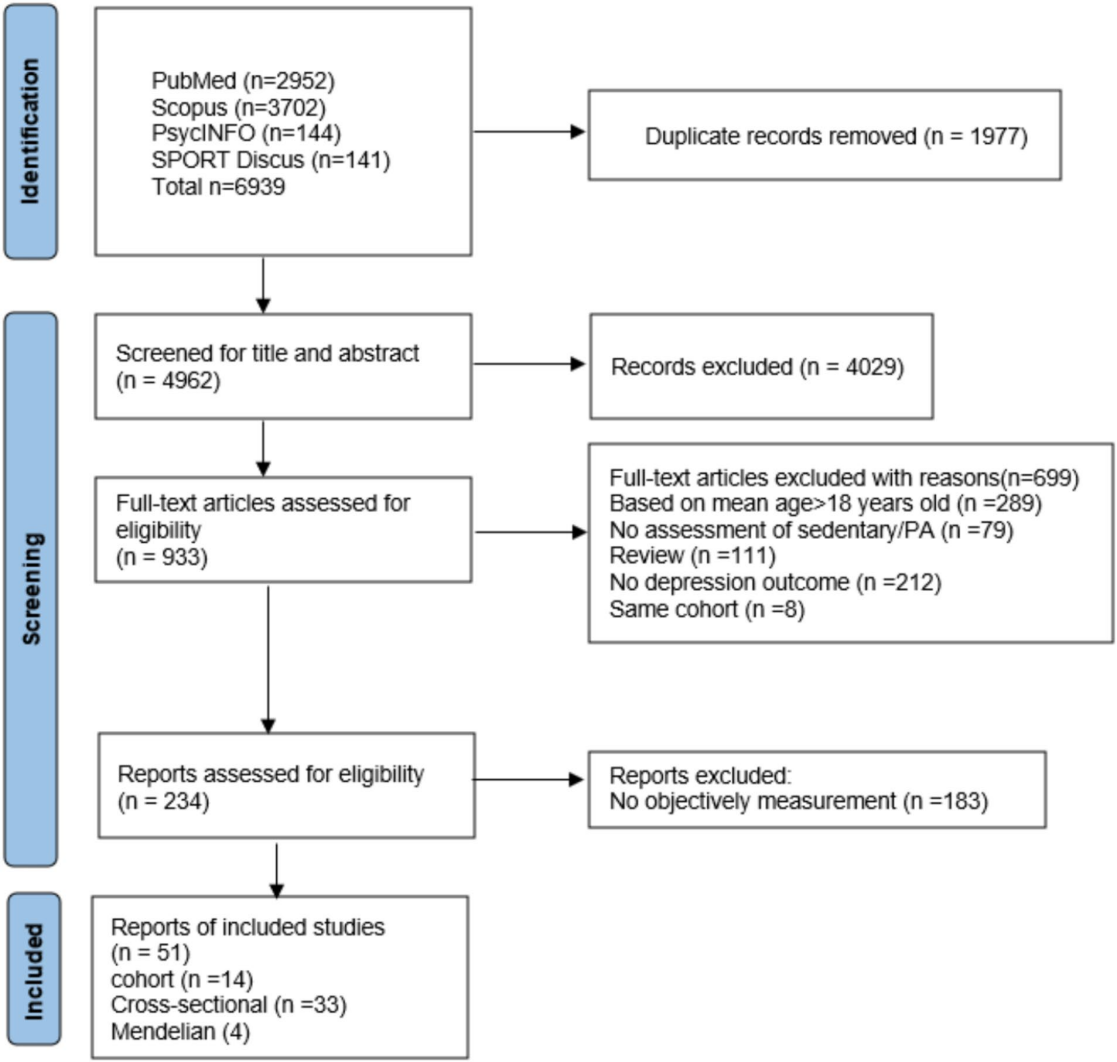
PRISMA flow diagram for the identification of included studies.

Initially, we identified 6,939 articles, with 1977 remaining after deduplication. Following title and abstract screening, 234 articles underwent full-text eligibility assessment. An additional article was included post-reference check, culminating in a final analysis of 51 articles, encompassing 14 cohort studies (Blodgett et al., 2023; Chan et al., 2022; Chan et al., 2023; Herbolsheimer et al., 2018; Ho et al., 2022; Hsueh et al., 2020; Hussenoeder et al., 2023; King et al., 2022; Konopka et al., 2022; Ku et al., 2018; Okely et al., 2019; Mossavar-Rahmani et al., 2023; Siwa et al., 2023; Zhang et al., 2021), 33 cross-sectional studies (Araki et al., 2022; Asai et al., 2018; Del Pozo Cruz et al., 2020; Bae et al., 2023; Biddle et al., 2021; Bustamante et al., 2013; Casals et al., 2024; Chen et al., 2023; Dennison et al., 2021; Dhakal et al., 2023; Eriksson et al., 2020; Felez-Nobrega et al., 2023; Heinz et al., 2022; Hollands et al., 2020; Hsiao et al., 2022; Kirschner et al., 2022; Larisch et al., 2020; Lee et al., 2014; Li et al., 2022; Loprinzi, 2013; Loprinzi and Mahoney, 2014; Maher et al., 2018; McKercher et al., 2009; Michalak et al., 2022; Morres et al., 2019; Park et al., 2024; Rethorst et al., 2017; Song et al., 2012; Tully et al., 2020; Vallance et al., 2011; Wemeck et al., 2022; Yasunaga et al., 2018; Zhou et al., 2024) and 4 Mendelian randomization study (Ba et al., 2024; Casanova et al., 2023; Choi et al., 2019; Iob et al., 2023). A visualization of the article selection process is shown in Figure 1.

Supplementary Table S5 details the characteristics of 51 studies, encompassing 1,318,687 participants across 14 cohort studies (*n* = 134,047), 33 cross-sectional studies (n = 253,221), and Mendelian randomization studies (*n* = 812,245). These studies were conducted in Europe (*n* = 7: UK, Germany, Poland, Spain, Sweden, Greece), Asia (*n* = 4: Taiwan, China, South Korea, Japan), Oceania (*n* = 1: Australia), North America (*n* = 1: USA), and South America (*n* = 1: Brazil). Participants’ mean age spanned from 18.0 to 86 years, with female representation ranging from 0 to 81.4%. Depression rates across studies varied between 3.2 and 48.8% (Supplementary Table S6). The methodological quality of the studies is detailed in Supplementary Table S8, with 33 out of 51 studies classified as high quality and one study as low quality.

### 3.2 Measurement method

Supplementary Table S7 provides a summary of the measurement instruments utilized, encompassing accelerometers such as ActiGraph, MoveMonitor, Fitmit Charge, GENEActiv Original, StepWatch™, McRoberts, Actiwatch 2, GENEActiv, Active style ProHJA-350IT, Vitamove, and Yamax Digiwalker. The study employed five accelerometer types, four pedometer types, and three additional devices worn on the hip, wrist, thigh, and triceps. Various cut-off values were applied to evaluate i-SB and i-PA, with i-SB ranging from 1,040 to 2,689 CPM or 1.51 to 2.99 METs, and MVPA thresholds set at >1,040 and ≥ 2,690 CPM or ≥ 3.00 METs. Data collection spanned a minimum of four consecutive days, with the majority of studies extending this period to seven consecutive days.

### 3.3 Results for instrumented sedentary behavior and physical activity

#### 3.3.1 Sedentary behavior and depression

**Figure 2:**
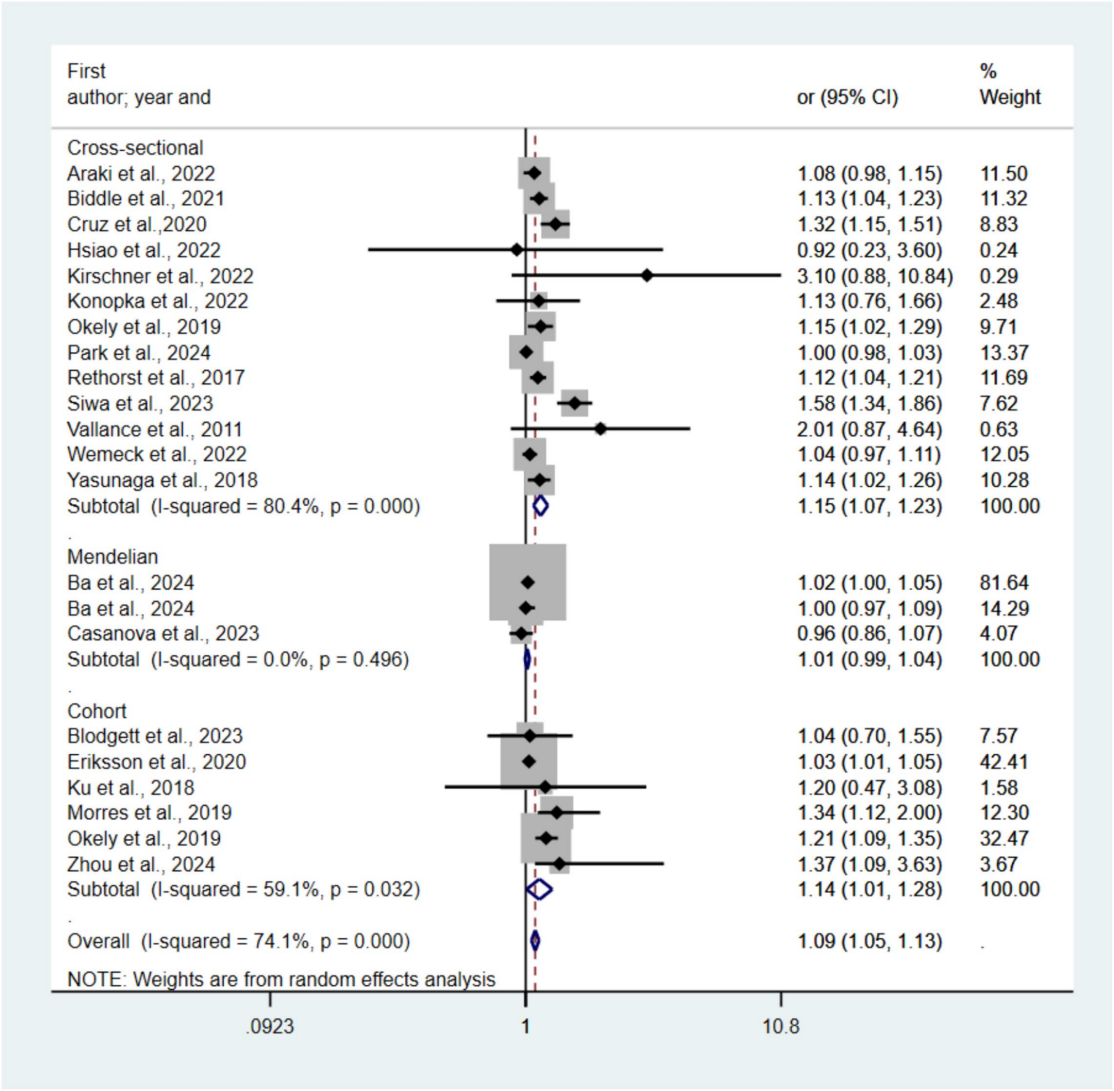
Forest plots of the association between i-SB and risk of depression.

Supplementary Table S6 presents an overview of the association between i-SB, i-PA, and depression. Figure 2 presents forest plots depicting the link between i-SB and depression risk across 21 studies. The highest i-SB category exhibited an OR of 1.09 (95% CI 1.05–1.13, *p* < 0.05, *I*^2^ = 93.8) relative to the lowest category, as determined by a pooled random-effects model. The OR for cross-sectional studies is 1.15 (95% CI 1.07–1.23), for cohort studies is 1.14 (95% CI 1.01–1.28), and for Mendelian randomization method studies is 1.01 (95% CI 0.99–1.04). The funnel plots (Supplementary Figure SA) indicated no statistically significant publication bias (Egger’s test *p* = 0.18). Tully et al. (2020) was excluded after sensitivity analysis.

#### 3.3.2 Light physical activity and depression

**Figure 3:**
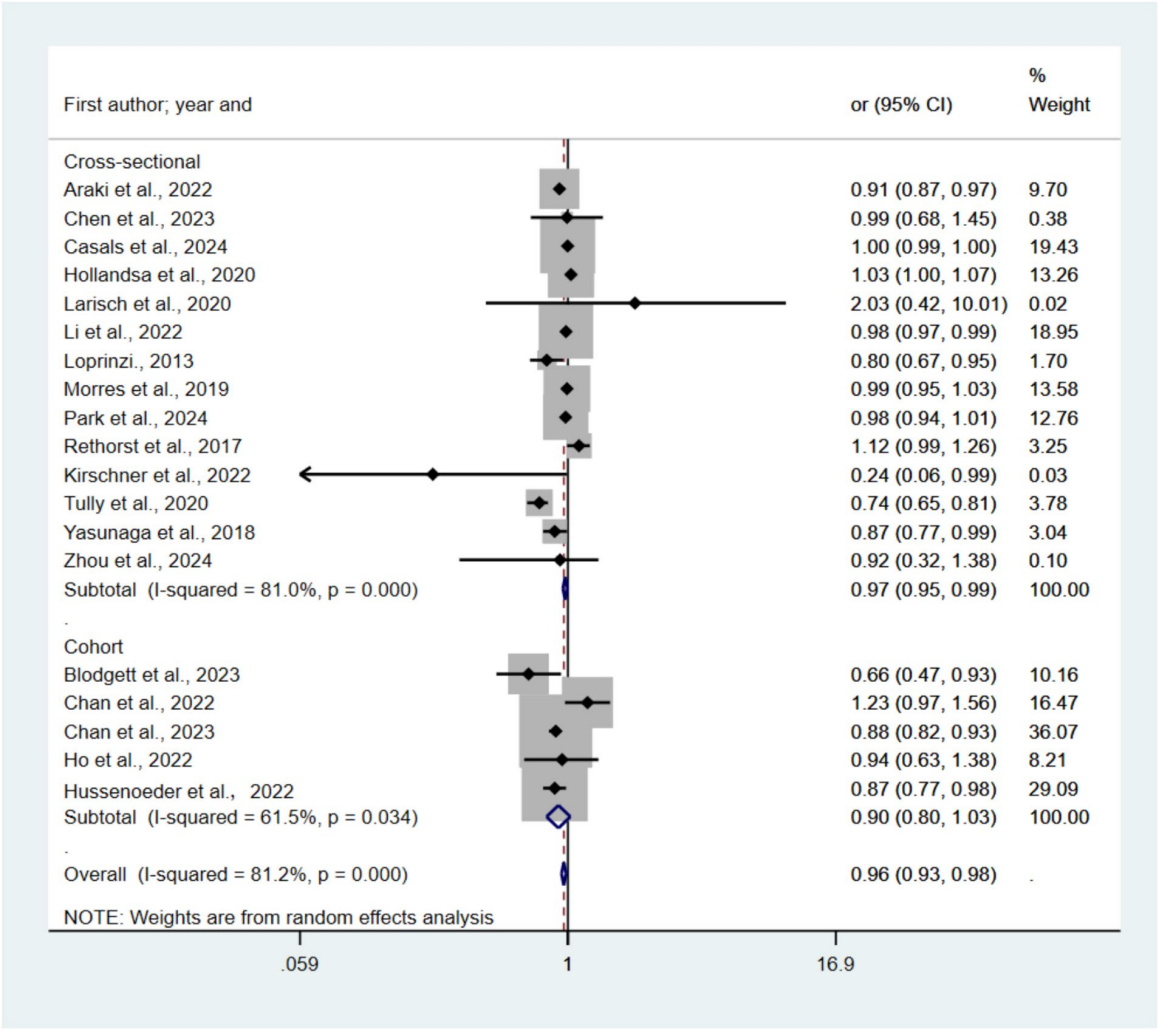
Forest plots of the association between LPA and risk of depression.

Figure 3 presents forest plots depicting the correlation between LPA and depression risk across nineteen included studies. Compared with the lowest LPA category, the pooled random-effects model resulted in an OR of 0.96 (95% CI 0.93–0.98, *p* < 0.05, *I*^2^ = 81.2) for the highest category. The OR for cross-sectional studies is 0.97 (95% CI 0.95–0.99), and for cohort studies is 0.90 (95% CI 0.93–0.98). The funnel plots (see Supplementary Figure SB) indicated no statistically significant publication bias (Egger’s test *p* = 0.31). Sensitivity analysis showed that excluding any single study did not substantially alter the pooled effect estimates.

#### 3.3.3 Moderate-to-vigorous physical activity and depression

**Figure 4:**
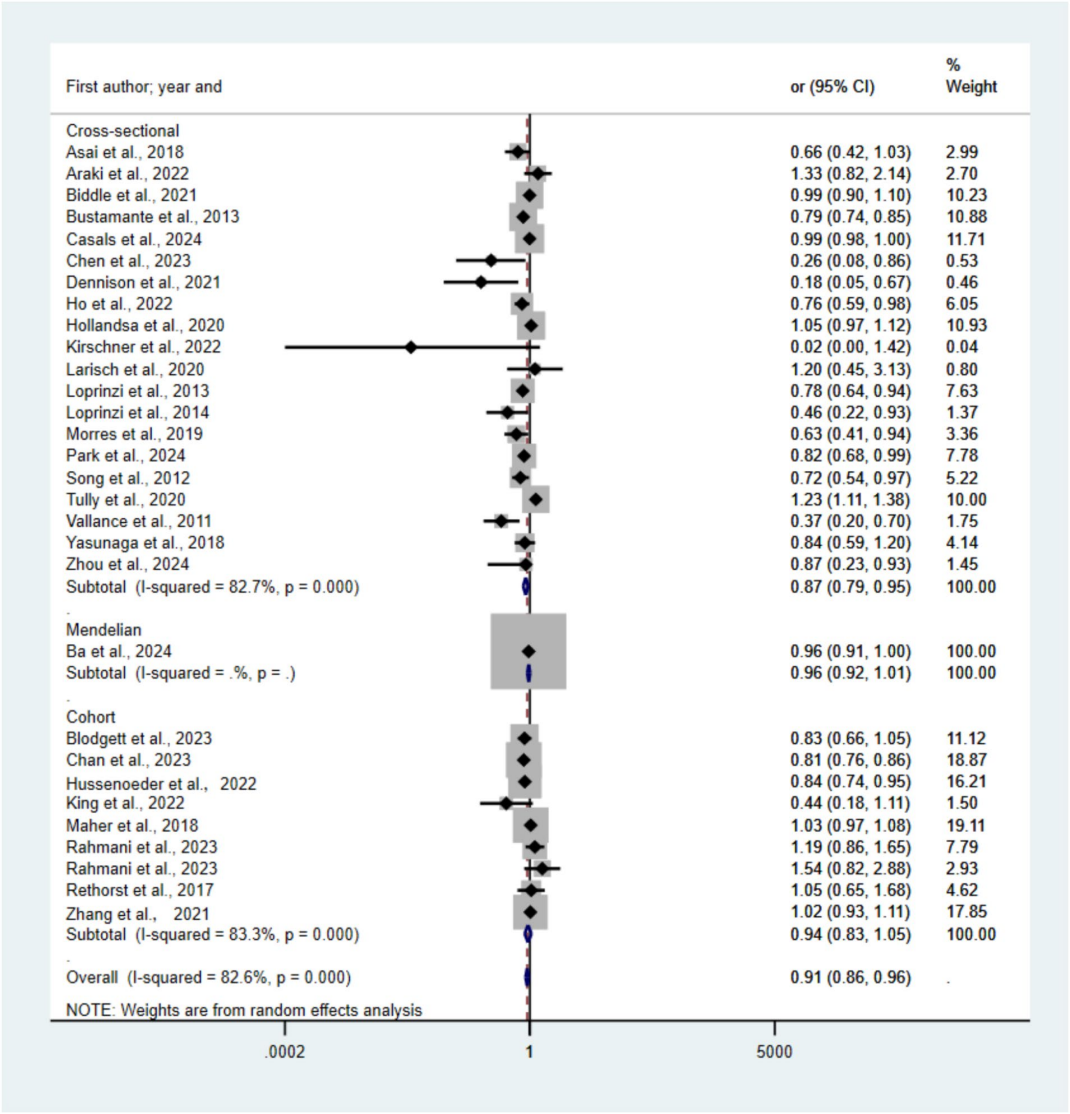
Forest plots of the association between MVPA and risk of depression.

Figure 4 presents forest plots depicting the link between MVPA and depression risk across 30 included studies. Compared with the lowest MVPA category, the pooled random-effects model resulted in an OR of 0.91 (95% CI 0.86–0.96, *p* < 0.05, *I*^2^ = 82.6) for the highest category. The OR for cross-sectional studies is 0.87 (95% CI 0.79–0.95), for cohort studies is 0.94 (95% CI 0.83–1.05), and for Mendelian randomization method studies is 0.96 (95% CI 0.92–1.01). The funnel plots (see Supplementary Figure SC) indicated no statistically significant publication bias (Egger’s test *p* = 0.40). Konopka et al. (2022) was excluded after sensitivity analysis.

#### 3.3.4 Total physical activity and depression

**Figure 5:**
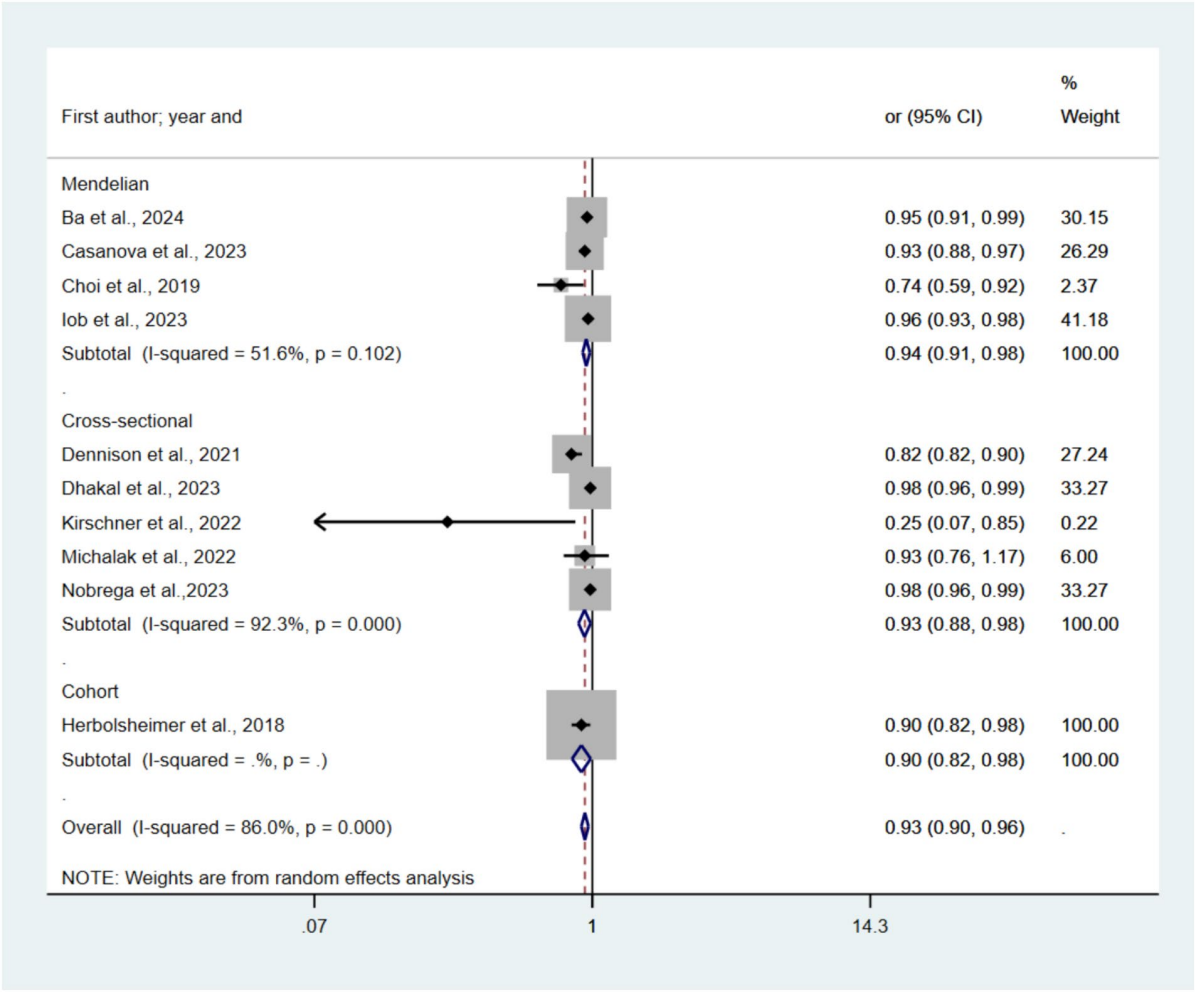
Forest plots of the association between TPA and risk of depression.

Figure 5 presents forest plots depicting the relationship between TPA and depression risk across 30 studies. The pooled random-effects model revealed an OR of 0.93 (95% CI 0.90–0.96, *p* < 0.05, *I*^2^ = 86.0%) for the highest TPA category compared to the lowest. Subgroup analyses yielded ORs of 0.93 (95% CI 0.88–0.98) for cross-sectional studies, 0.90 (95% CI 0.82–0.98) for cohort studies, and 0.94 (95% CI 0.91–0.98) for Mendelian randomization studies. Statistically significant publication bias was detected via funnel plot and Egger’s test (*p* = 0.00). Sensitivity analysis, with individual study exclusion, showed no significant alterations in the combined effect size.

#### 3.3.5 Step and depression

**Figure 6:**
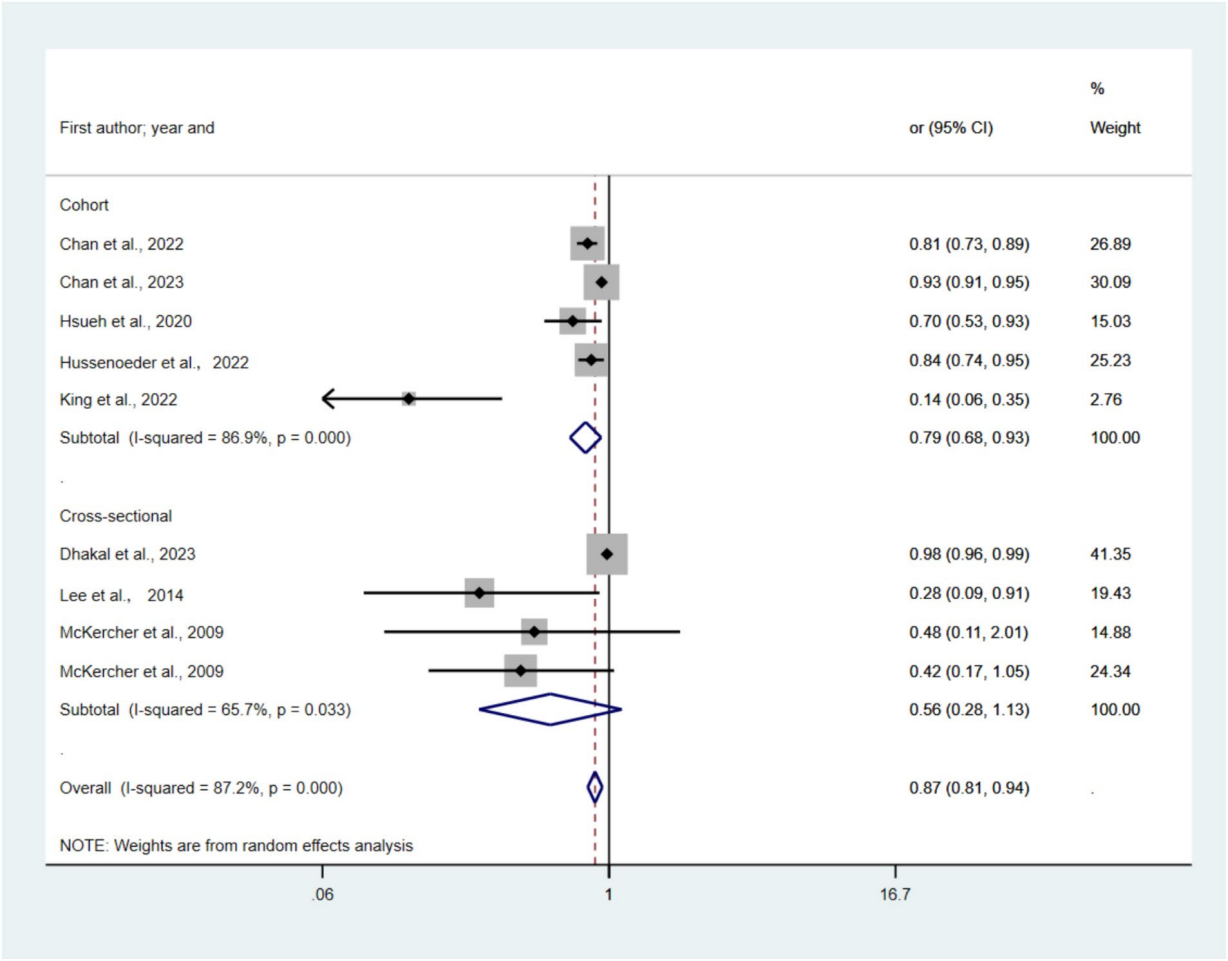
Forest plots of the association between Daily Step and risk of depression.

Figure 6 presents forest plots depicting the link between TPA and depression risk across nine studies. The pooled random-effects model indicated a significantly lower odds ratio (OR) of 0.87 (95% CI: 0.81–0.94, *p* < 0.05, I2 = 87.2%) for the highest TPA category compared to the lowest. The stratified analysis revealed ORs of 0.56 (95% CI: 0.28–1.13) for cross-sectional studies and 0.79 (95% CI, 0.68–0.93) for cohort studies. Statistically significant publication bias was detected using both funnel plot analysis (Supplementary Figure SE) and Egger’s test (*p* = 0.08). Sensitivity analysis, with individual study exclusion, showed no significant alterations in the combined effect size.

### 3.4 Subgroup analysis

Table 1 presents the outcomes of subgroup analyses, assessing the stability of pooled results and identifying potential heterogeneity sources. For MVPA, heterogeneity was significantly associated with study design and depression scales, suggesting their impact on pooled ORs. In the case of SB, heterogeneity was significantly linked to study design and BMI-related confounding factors, indicating their influence on pooled ORs.

**Table 1:** Results of the subgroup analysis.

Subgroup		MVPA					SB				
		*N*	OR (95% CI)	*I*^2^ (%)	*p* ^a*^	*P* ^b*^	*N*	OR (95% CI)	*I*^2^ (%)	*p* ^a*^	*P* ^b*^
Age	Adult	12	0.77 (0.67,0.87)	84.70	0.00	0.00	8	1.03 (1.00,1.06)	9.96	0.21	0.03
	Older	6	0.99 (0.94,1.03)	10.91	0.00		8	1.07 (1.03,1.11)	27.33	0.03	
	Mix	12	0.87 (0.79,0.96)	73.53	0.00		6	1.15 (1.06,1.24)	54.54	0.00	
Depression scales	CES	10	0.93 (0.85,1.01)	77.55	0.00	0.09	5	1.08 (0.72,1.43)	98.58	0.16	0.27
	GDS	6	0.99 (0.95,1.02)	10.65	0.00		5	1.06 (1.01,1.12)	33.70	0.37	
	PHQ	5	0.67 (0.53,0.81)	55.4	0.00		6	1.18 (0.96,1.24)	52.85	0.00	
	Others	9	0.81 (0.68,0.94)	79.94	0.00		7	1.15 (1.05,1.25)	74.06	0.00	
Confounding factor											
BMI	Yes	21	0.82 (0.63,0.89)	83.66	0.00	0.23	18	1.07 (1.03,1.12)	49.28	0.00	0.02
	No	9	0.96 (0.92,1.00)	22.58	0.00		4	1.29 (1.09,1.49)	32.42	0.01	
SES	Yes	15	0.88 (0.68,0.98)	82.7	0.00	0.50	9	1.07 (1.03,1.1)	66.28	0.00	0.83
	No	15	0.91 (0.87,0.95)	47.32	0.00		13	1.05 (1.03,1.10)	37.42	0.01	
Ethnicity	Yes	11	0.82 (0.73,0.92)	82.24	0.00	0.09	4	1.15 (0.97,1.42)	13.54	0.19	0.01
	No	19	0.91 (0.67,0.99)	55.36	0.00		18	1.16 (1.04,1.28)	51.92	0.19	
baseline disease	Yes	14	0.86 (0.65,0.94)	84.19	0.00	0.64	11	1.06 (1.02,1.10)	57.77	0.00	0.42
	No	16	0.88 (0.84,0.93)	46.50	0.00		11	1.09 (1.03,1.15)	43.88	0.00	

CS, cross-sectional study; SES, socioeconomic status; P^a^ and P^b^ represent heterogeneity within and between subgroups, respectively.

Significant within-subgroup heterogeneity in the studies on MVPA was found in all subgroups, indicating that between-group heterogeneity or other unknown factors may influence heterogeneity. Regarding i-SB, significant heterogeneity was also found in the SB subgroups, except for the Adult, CES, GDS, and Non-Adjusted Ethnicity groups. The findings indicate potential influences on the pooled odds ratios (ORs) of the subgroups by these factors. The relationship between i-PA, i-SB, and depression was inconsistent across most analyses. The high heterogeneity among the included studies may be attributed to the large number of included studies and disparities in the study design, depression ascertainment, and adjustment variables.

### 3.5 The impact of sedentary behavior and physical activity on depression after mutual adjustment

Regarding SB, seven articles adjusted for time spent in PA, and 14 articles with no adjustment for PA were identified. The pooled random-effects model resulted in an OR with adjusted PA of 1.02 (95% CI 1.00–1.04, *p* = 0.53 > 0.05, *I*^2^ = 0), while the OR for no PA adjusted was 1.20(95% CI 1.08–1.33, *p* = 0.00 < 0.05, *I*^2^ = 95.2%) (see Supplementary Figure SH). Regarding MVPA, four articles adjusted for time spent in SB, and 25 articles did not adjust for SB. The pooled random-effects model resulted in an OR with an adjusted SB of 0.63 (95% CI 0.44–0.89, *p* = 0.00 < 0.05, *I*^2^ = 92.1%), while the OR with no SB adjusted was 0.92 (95% CI 0.87–0.98, *p* = 0.00 < 0.05, *I*^2^ = 82.9%) (see Supplementary Figure SG).

### 3.6 Dose–response relationship

**Figure 7:**
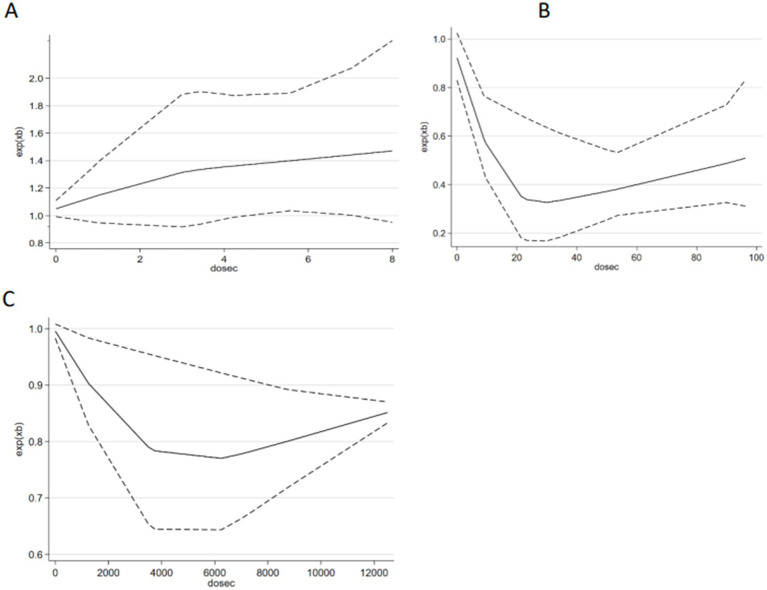
The dose–response relationship between i-SB, MVPA, daily step and depression risk.

For SB, we found a linear association relationship between SB and the risk of depression (*p*_nonlinearity_ = 0. 30) (Figure 7A). The risk of depression increased by 4% for every additional hour of SB per day SB (*p* < 0.01; OR = 1.04, 95% CI 0.90–1.19). For MVPA, we found a nonlinear association relationship between MVPA and depression (*p*_nonlinearity_ = 0.0077) (Figure 7B). When MVPA was <35 min/day, the risk of depression decreased by 20% for every additional 5 min/day of MVPA (*p* < 0.01; OR = 0.80, 95% CI 0.60–0.94). When MVPA was >35 min/day, the risk of depression increased by 16% for every additional 5 min/day of MVPA (*p* < 0.01; OR = 1.16, 95% CI 0.92–1.56). For steps per day, we found a nonlinear association relationship between steps per day and depression (*p*_nonlinearity_ = 0.0016) (Figure 7C). When steps per day was <4,000 steps/day, the risk of depression decreased by 9% for every additional 1,000 steps/day of MVPA (*p* < 0.01; OR = 0.91, 95% CI 0.81–0.99). When steps per day was >4,000 steps/day, the risk of depression decreased by 8% for every additional 1,000 steps/day of MVPA (*p* < 0.01; OR = 1.08, 95% CI 0.96–1.22).

## 4 Discussion

To the best of our knowledge, this is the first systematic review and dose–response meta-analysis of the association between instrumented measures of SB, PA, and depression among adult and older populations. This meta-analysis showed that both higher i-SB levels and lower i-PA levels were associated with a higher depression risk. For SB, LPA, MVPA, TPA, and step, the effect size OR values were 1.09 (95% CI 1.05–1.13), 0.96 (95% CI 0.93–0.98), 0.91 (95% CI 0.86–0.96), 0.93 (95% CI 0.90–0.96), and 0.87 (95% CI 0.81–0.94). The lowest OR was observed for the step group. The study also reported mutually adjusted ORs for PA and SB. Meta-regression analyses confirmed significant dose–response associations between SB, MVPA, daily steps, and depression risk.

The OR of 1.16 (95% CI 1.08–1.24) found in this study is lower than those reported in previous meta-analyses examining the link between self-reported sedentary behavior and depression [1.42, 95% CI: 1.22–1.67 (Zhou et al., 2023); 1.28, 95% CI 1.17–1.39 (Wang et al., 2019), 1.25, 95% CI 1.16–1.35 (Zhai et al., 2015)]. The OR for physical activity (PA) in this study, 0.87 (95% CI 0.81–0.94), closely aligns with prior meta-analyses examining the link between self-reported PA and depression, particularly when contrasting inactive with active adults and older populations [0.90, 95% CI: 0.83–0.98 (Schuch et al., 2019); 0.83, 95% CI: 0.78–0.98 (Guo et al., 2022)]. SB is often underestimated and PA is often overestimated when using self-reported measures (Ryan et al., 2018). Prior meta-analyses and observational studies may have been affected by this, potentially affecting the precision of the depression risk estimation. Recently, Gianfredi et al. (2020) conducted a meta-analysis of the associations between objectively measured PA and incident and prevalent depression, including 37,408 participants, with the overall effect size of depression for the highest vs. the lowest level of PA being −1.16 (95% CI −1.41 to −0.91). In contrast to the previous meta-analysis, which encompassed studies with chronic obstructive pulmonary disease or cancer patients and middle-aged to elderly populations, our analysis focused exclusively on healthy populations, excluding those with patient groups. Furthermore, our study utilized objective metrics for physical activity and sedentary behavior to assess depression risk, offering a more precise and targeted analysis. Furthermore, the ORs from Mendelian randomization studies based on a large sample were 1.01(0.99, 1.04) for i-SB and 0.94(0.91,0.98) for i-PA, which are both more negative than cohort and cross-sectional studies. Therefore, future research should cautiously interpret the association of PA and SB with depression.

Subgroup analysis revealed significant age-related heterogeneity, suggesting age moderates the relationship between physical activity, sedentary behavior and depression among adult and older populations. In elderly populations, these associations were non-significant, with higher ORs than adults, potentially indicating a weakening relationship with age. These findings contrast with Dishman et al. (2021), who found no age difference among the adult population. Gender stratification was limited due to insufficient sex-disaggregated data, and findings from the included study on gender moderation were inconsistent. Hsueh et al. (2020) and others reported reduced depression risk with increased physical activity only in men (OR: 0.75, 95% CI: 0.62–0.91), while Song et al. (2012) found a higher risk only in women (OR: 1.47, 95% CI: 1.12–1.93). Several studies reported non-significant differences in results for sex subgroups, including Hussenoeder et al. (2023). Wemeck et al. (2022) noted potential gender differences, opposing prior findings of stronger associations in women. Further research is needed to clarify these discrepancies and explore underlying mechanisms.

Another main finding in this study was that after adjusting for PA, i-SB did not significantly correlate with an increased risk of depression (1.02:95% CI 1.00–1.04, *p* = 0.53 > 0.05). The study indicates that PA could reduce the depressive risk associated with SB. Even after accounting for SB, MVPA with a lower OR remained significantly linked to a decreased risk of depression (OR = 0.63; 95% CI, 0.44–0.89; *p* < 0.05), suggesting that SB does not affect PA’s protective role against depression. These findings are consistent with those of a previous observational study on the risk of depression among adolescents (De Faria et al., 2022) and a meta-analysis on all-cause mortality risk (Rojer et al., 2020). In brief, the link between PA and the risk of depression was independent of SB, and the link between SB and the risk of depression was not independent of PA. The potential mechanism may be that PA promotes health holistically from physiological, psychological, and social perspectives. SB primarily impacts healthy lifestyles in the physiological dimension. A previous study indicated that sufficient PA is significantly associated with lower odds of depressive symptoms across low, moderate, and high levels of SB, suggesting that PA may play a protective role against depressive symptoms in adults even with prolonged SB (Liao et al., 2015). It should also be considered that PA for older adults yields benefits that extend beyond depression, such as the maintenance of functional independence, which might in turn contribute to a reduced likelihood of depression (Du et al., 2015).

The current research primarily employed two objective measurement tools: accelerometers and pedometers, irrespective of the specific models or wear locations. The overall heterogeneity may be attributed to improper installation of measurement components and a lack of standardization among various measurement devices. This problem has also been noted in previous studies, including one conducted by Gianfredi et al. (2020). Montoye et al. (2016) conducted a study that contrasted the accuracy of accelerometers placed on the hips, thighs, and wrists. These results suggest that wrist-worn devices are more sensitive and specific than hip-worn devices for assessing SB and LPA. The device worn on the thigh demonstrated maximum specificity and sensitivity. Therefore, To enable rigorous comparison across studies, it is imperative to use standardized methods and ensure the calibration of devices for evaluating i-SB and i-PA. The comparison of different studies is complicated by the absence of a standardized definition for the assessment of SB and PA, leading to the utilization of varying cut-off values and devices, which are significant sources of heterogeneity. Therefore, to facilitate the comparison of various studies, it is imperative to adopt standardized methodologies and ensure the calibration of different devices when evaluating SB and PA in future research.

Although the exact mechanisms underlying the impact of SB on the adult and older population’s mental health are not fully understood, various possibilities have been suggested. SB, when experienced in isolation, can evoke a sense of loneliness and hence have a detrimental effect on mental well-being (Hoare et al., 2016). Furthermore, the media’s transmission of cultural signals can influence other behaviors associated with mental health, such as eating disorders and aggressive conduct (Primack et al., 2009). Media overexposure is a common occurrence throughout the day and night. Throughout the day, engaging in screen-based activities may replace participation in more beneficial and/or physically active activities, including PA and face-to-face conversation (Ohannessian, 2009). Given this lack of information, research specifically examining the neurological underpinnings of these individuals is particularly intriguing.

Our research holds substantial implications for public mental health. It is imperative to quantify the relationship between internet-based sedentary behavior (i-SB) and internet-based physical activity (i-PA) with depression and to compare the effect sizes for i-SB and i-PA in this meta-analysis to facilitate optimal risk management of physical activity in adults and older populations. It is crucial to clarify this association because international standards for PA (150 min of moderate-intensity PA or 75 min of vigorous-intensity PA) are increasingly emphasizing the need to reduce sedentary time and screen use (WHO, 2020). Our findings underscore the significance of mitigating SB and enhancing PA for mental health. The meta-analysis indicates a bidirectional link between depressive symptoms and both PA and SB, implying that mental health surveillance and support could foster healthier lifestyle decisions. Incorporating structured PA into therapeutic regimens may prove advantageous for individuals experiencing depressive symptoms. Ultimately, while advocating for PA and curbing SB is essential for a balanced lifestyle across all age groups, these measures alone may not suffice to prevent depression. It is advisable to adopt a comprehensive approach that includes early intervention, tactics tailored to each gender, and integration with broader mental health assistance.

Our meta-analysis has several strengths. Primarily, the use of exclusively objective measures of PA and SB represents a strength of this review, as questionnaires may not capture unstructured PA, and adults and older populations are susceptible to overreporting PA and underreporting SB (Ryan et al., 2018; Dyrstad et al., 2014). This study included a large number of participants, with a total of 1,318,687. A key merit of this study is the precise measurement of the relationship between SB, PA, and depression, utilizing objective tools such as accelerometers and pedometers. The analyses, which incorporated cohort studies with diverse follow-up periods, suggest a minimal likelihood of reverse causation. This study incorporated Mendelian randomization research methods to further elucidate the causal relationships between related variables. Furthermore, we performed a conventional binary and dose–response meta-analysis to investigate the impact of these two factors on depression in adults and older populations. This technique effectively utilizes all available data and yields robust statistical results.

The study’s findings should be interpreted with caution due to several limitations. Firstly, the meta-analysis exhibited significant heterogeneity, likely attributable to the diverse studies included. Secondly, the absence of a standardized approach for evaluating i-SB and i-PA across studies hinders direct comparison, as it involves disparate cut-off values, measurement devices, wearing positions, durations, and analytical methods. Lastly, the 33 cross-sectional studies do not permit the establishment of causal inferences, as the relationships cannot be definitively determined. These studies are not equipped to ascertain causal relationships. Consequently, these findings necessitate cautious interpretation. To explore the interplay between PA, SB, and depression risk in adults and older populations, further longitudinal and interventional research is imperative.

## 5 Conclusion

The association between i-SB and the risk of depression was not consistent with the results of previous self-reported studies. The link between MVPA and the risk of depression was independent of i-SB, whereas the link between i-SB and the risk of depression was not independent of i-PA. We found a linear relationship between SB and the risk of depression and a nonlinear relationship between MVPA or steps per day and depression. The study underscores the necessity of advocating physical activity and curtailing sedentary behavior in forthcoming healthy lifestyle recommendations for adult and older populations.

## Data Availability

The original contributions presented in the study are included in the article/Supplementary material, further inquiries can be directed to the corresponding author/s.
