# Supplementary material for: Sedentary behavior and physical activity are associated with risk of depression among adult and older populations: a systematic review and dose–response meta-analysis
Source: Front Psychol. 2025 Mar 17;16:1542340. doi: 10.3389/fpsyg.2025.1542340 (PMC11955711; doi:10.3389/fpsyg.2025.1542340)
Supplement: Supplementary file 1 [file Table_1.DOCX]

# Supplementary Material Table S1-8.

# Full search strategy (conducted on 15-05-2024)

## **Table S.1 Search strategy in PubMed (2024 MAY 15th)**

| **#** | Query | Results |
| --- | --- | --- |
| **#7** | #3 AND #4 | \| **345** \| \| --- \| |
| **#4** | ((((((((accelerometry[MeSH Terms]) OR (accelerometer)) OR (Acceleromet)) OR (pedometer)) OR (Monitoring, Physiologic)) OR (Monitoring, Ambulatory)) OR (actigraphy)) OR (objectively measured)) OR (Instrumented measure) | **1,424,642** |
| **#3** | #1 AND #2 | **1,730** |
| **#2** | "Exercise"[MeSH Terms] OR ("Exercises"[Title/Abstract] OR "physical activity"[Title/Abstract] OR "activities physical"[Title/Abstract] OR "activity physical"[Title/Abstract] OR "physical activities"[Title/Abstract] OR "exercise physical"[Title/Abstract] OR "exercises physical"[Title/Abstract] OR "physical exercise"[Title/Abstract] OR "physical exercises"[Title/Abstract] OR "acute exercise"[Title/Abstract] OR "acute exercises"[Title/Abstract] OR "exercise acute"[Title/Abstract] OR "exercises acute"[Title/Abstract] OR "exercise isometric"[Title/Abstract] OR "exercises isometric"[Title/Abstract] OR "isometric exercises"[Title/Abstract] OR "isometric exercise"[Title/Abstract] OR "exercise aerobic"[Title/Abstract] OR "aerobic exercise"[Title/Abstract] OR "aerobic exercises"[Title/Abstract] OR "exercises aerobic"[Title/Abstract] OR "exercise training"[Title/Abstract] OR "exercise trainings"[Title/Abstract] OR "training exercise"[Title/Abstract] OR (("education"[MeSH Subheading] OR "education"[All Fields] OR "Training"[All Fields] OR "education"[MeSH Terms] OR "train"[All Fields] OR "train s"[All Fields] OR "trained"[All Fields] OR "training s"[All Fields] OR "Trainings"[All Fields] OR "trains"[All Fields]) AND "Exercise"[Title/Abstract])) OR ("sedentary behavior"[MeSH Terms] OR ("behavior sedentary"[Title/Abstract] OR "sedentary behaviors"[Title/Abstract] OR "sedentary lifestyle"[Title/Abstract] OR "lifestyle sedentary"[Title/Abstract] OR "physical inactivity"[Title/Abstract] OR "inactivity physical"[Title/Abstract] OR "lack of physical activity"[Title/Abstract] OR "sedentary time"[Title/Abstract] OR "sedentary times"[Title/Abstract] OR "time sedentary"[Title/Abstract])) | **458,463** |
| **#1** | "depressive disorder"[MeSH Terms] OR "Depression"[MeSH Terms] OR "depressive symptoms"[Title/Abstract] OR "depressive symptom"[Title/Abstract] OR "symptom depressive"[Title/Abstract] OR "emotional depression"[Title/Abstract] OR "depression emotional"[Title/Abstract] | **291,745** |
| **Table S.2 Search strategy in Scopus (2024 MAY 15th)** | |  |
| # | Query | Results |
| **S4** | S1 AND S2 AND S3 | 702 |
| **S3** | All field: "Accelerometry" or "Accelerometer" or "Acceleromet" or "Pedometer" or "Monitoring, Physiologic" or "Monitoring, Ambulatory" or "Actigraphy" or "objectively measured" or "Instrumented measures" | **161,579** |
| **S2** | Article title, Abstact, keywords: "Physical Activity" or "Activities, Physical" or "Activity, Physical" or "Physical Activities" or "Exercise, Physical" or "Exercises, Physical" or "Physical Exercise" or "Physical Exercises" or "Acute Exercise" or "Acute Exercises" or "Exercise, Acute" or "Exercises, Acute" or "Exercise, Isometric" or "Exercises, Isometric" or "Isometric Exercises" or "Isometric Exercise" or "Exercise, Aerobic" or "Aerobic Exercise" or "Aerobic Exercises" or "Exercises, Aerobic" or "Exercise Training" or "Exercise Trainings" or "Training, Exercise" or "Trainings, Exercise" or "Sedentary Behavior" or "Behavior, Sedentary" or "Sedentary Behaviors" or "Sedentary Lifestyle" or "Lifestyle, Sedentary" or "Physical Inactivity" or "Inactivity, Physical" or "Lack of Physical Activity" or "Sedentary Time" or "Sedentary Times" or "Time, Sedentary" | **402,155** |
| **S1** | Article title, Abstact, keywords: "Depression" or "Depressive Symptoms" or "Depressive Symptom" or "Symptom, Depressive" or "Emotional Depression" or "Depression, Emotional" | **944,273** |
| **Table S.3 Search strategy in PsycINFO via EBSCO (2024 MAY 15th)** | |  |
| # Query | | Results |

| **S4** | S1 AND S2 AND S3 | **156** |
| --- | --- | --- |
| **S3** | TX All Text (Accelerometry* or "Accelerometer*" or "Acceleromet*" or "Pedometer*" or "Monitoring, Physiologic" or "Monitoring, Ambulatory" or "Actigraphy*" or "objectively measured" or "Instrumented measures") | **69,937** |
| **S2** | TI (Physical Activity* or "Activities, Physical" or "Activity, Physical" or "Physical Activities*" or "Exercise, Physical" or "Exercises, Physical" or "Physical Exercise" or "Physical Exercises" or "Acute Exercise" or "Acute Exercises" or "Exercise, Acute" or "Exercises, Acute" or "Exercise, Isometric" or "Exercises, Isometric" or "Isometric Exercises" or "Isometric Exercise" or "Exercise, Aerobic" or "Aerobic Exercise" or "Aerobic Exercises" or "Exercises, Aerobic" or "Exercise Training" or "Exercise Trainings" or "Training, Exercise" or "Trainings, Exercise" or "Sedentary Behavior" or "Behavior, Sedentary" or Sedentary Behaviors* or "Sedentary Lifestyle" or "Lifestyle, Sedentary" or "Physical Inactivity" or "Inactivity, Physical" or "Lack of Physical Activity" or "Sedentary Time" or "Sedentary Times" or "Time, Sedentary"  ) OR AB (Physical Activity* or "Activities, Physical" or "Activity, Physical" or "Physical Activities*" or "Exercise, Physical" or "Exercises, Physical" or "Physical Exercise" or "Physical Exercises" or "Acute Exercise" or "Acute Exercises" or "Exercise, Acute" or "Exercises, Acute" or "Exercise, Isometric" or "Exercises, Isometric" or "Isometric Exercises" or "Isometric Exercise" or "Exercise, Aerobic" or "Aerobic Exercise" or "Aerobic Exercises" or "Exercises, Aerobic" or "Exercise Training" or "Exercise Trainings" or "Training, Exercise" or "Trainings, Exercise" or "Sedentary Behavior" or "Behavior, Sedentary" or Sedentary Behaviors* or "Sedentary Lifestyle" or "Lifestyle, Sedentary" or "Physical Inactivity" or "Inactivity, Physical" or "Lack of Physical Activity" or "Sedentary Time" or "Sedentary Times" or "Time, Sedentary"  ) | **154,841** |
| **S1** | TI (Depression* or "Depressive Symptoms" or "Depressive Symptom" or "Symptom, Depressive" or "Emotional Depression" or "Depression, Emotional" ) OR AB (Depression* or "Depressive Symptoms" or "Depressive Symptom" or "Symptom, Depressive" or "Emotional Depression" or "Depression, Emotional" ) | **453,366** |

## **Table S.4 Search strategy in SPORTDiscus via EBSCO (2024 MAY 15th)**

| # Query | Results |
| --- | --- |

| **S4** | S1 AND S2 AND S3 | **132** |
| --- | --- | --- |
| **S3** | TX All Text (Accelerometry* or "Accelerometer*" or "Acceleromet*" or "Pedometer*" or "Monitoring, Physiologic" or "Monitoring, Ambulatory" or "Actigraphy*" or "objectively measured" or "Instrumented measures") | **76,898** |
| **S2** | TI (Physical Activity* or "Activities, Physical" or "Activity, Physical" or "Physical Activities*" or "Exercise, Physical" or "Exercises, Physical" or "Physical Exercise" or "Physical Exercises" or "Acute Exercise" or "Acute Exercises" or "Exercise, Acute" or "Exercises, Acute" or "Exercise, Isometric" or "Exercises, Isometric" or "Isometric Exercises" or "Isometric Exercise" or "Exercise, Aerobic" or "Aerobic Exercise" or "Aerobic Exercises" or "Exercises, Aerobic" or "Exercise Training" or "Exercise Trainings" or "Training, Exercise" or "Trainings, Exercise" or "Sedentary Behavior" or "Behavior, Sedentary" or Sedentary Behaviors* or "Sedentary Lifestyle" or "Lifestyle, Sedentary" or "Physical Inactivity" or "Inactivity, Physical" or "Lack of Physical Activity" or "Sedentary Time" or "Sedentary Times" or "Time, Sedentary"  ) OR AB (Physical Activity* or "Activities, Physical" or "Activity, Physical" or "Physical Activities*" or "Exercise, Physical" or "Exercises, Physical" or "Physical Exercise" or "Physical Exercises" or "Acute Exercise" or "Acute Exercises" or "Exercise, Acute" or "Exercises, Acute" or "Exercise, Isometric" or "Exercises, Isometric" or "Isometric Exercises" or "Isometric Exercise" or "Exercise, Aerobic" or "Aerobic Exercise" or "Aerobic Exercises" or "Exercises, Aerobic" or "Exercise Training" or "Exercise Trainings" or "Training, Exercise" or "Trainings, Exercise" or "Sedentary Behavior" or "Behavior, Sedentary" or Sedentary Behaviors* or "Sedentary Lifestyle" or "Lifestyle, Sedentary" or "Physical Inactivity" or "Inactivity, Physical" or "Lack of Physical Activity" or "Sedentary Time" or "Sedentary Times" or "Time, Sedentary"  ) | **203,348** |
| **S1** | TI (Depression* or "Depressive Symptoms" or "Depressive Symptom" or "Symptom, Depressive" or "Emotional Depression" or "Depression, Emotional"  ) OR AB (Depression* or "Depressive Symptoms" or "Depressive Symptom" or "Symptom, Depressive" or "Emotional Depression" or "Depression, Emotional"  ) | **331,991** |

##

| **Table S.5 Characteristics of included cohorts** | | | | | | | |
| --- | --- | --- | --- | --- | --- | --- | --- |
| Author(year） | **Country** | **Cohort** | Age(years） | No of participants (women） | **PA measures** | **Wearing location** | **FU(years)** |
| **cohort studies** | | | | | | | |
| Blodgett et al., 2023 | UK | 1970 British Cohort Study | 46 | 16571(n/r) | MVPA;LPA;SB | the thigh | 2 years |
| Chan et al., 2022 | AU | 322 elderly people living in Australia | 75.5 | 322(62.1%) | Daily steps | the lower back | 2 years |
| Chan et al., 2023 | AU | the UK Biobank Resource | 62.0(7.85) | 72359(53.3%) | Steps per day | wrist | 7.4 years |
| Herbolsheimer et al., 2018 | DE | ActiFE Ulm study | 72.6 (5.0) | 334(39.2%) | PA | thigh | 3 years |
| Ho et al., 2022 | UK | UK Biobank | 37–73 years | 37327(n/r) | MVPA;LPA;VPA;SB | Wrist | 6.8 years |
| Hsueh et al., 2020 | TW | Taiwan's Kaohsiung City seniors, aged ≥65. | 74.52(6.12) | 285(54.4%) | Daily steps | waist | 2 years |
| Hussenoeder et al., 2022 | DE | 1451 participants | 55.0 (11.8) | 1451(52.1%) | Daily steps |  | 2 years |
| King et al., 2022 | USA | LABS-2 | 47 | 646(77.7%) | PA;SB;MVPA | ankle | 7 years |
| Konopka et al., 2022 | NL | The Maastricht Study | 60.1(8.0) | 2082(48.8%) | ST;LPA;HPA | the right thigh | 4 years |
| Ku et al., 2017 | TW | 285 community-dwelling older adults | 74.5 (6.1) | 285((54.4%) | LPA;MVPA;SB | waist | 2 years |
| Okely et al., 2019 | UK | Birth Cohort 1936; Birth Cohort 1950;Birth Cohort 1930s: | S1:78.97(0.44) S2:64.58(0.90) S3:83.40(0.62) | LBC1936:271 Twenty-07 1950s:309 Twenty-07 1930s:118 | SB | the thigh | 8 years |
| Rahmani et al., 2023 | USA | 16,415 Hispanic/Latino adults in four U.S. metro areas participated | 49.2(11.5) 46.9(9.2) | 3823(65.4%) 2009(53.1%) | SB;LPA;MVPA | hip | 7.64 years  7.83 years |
| Siwa et al., 2023 | PL | 320 focus person-partner adult dyads were enrolled | 43.86 | 640(50%) | SB;LPA;MVPA | dominant hip | 1.17 years |
| Zhang et al., 2021 | USA | 2871 from The CARDIA study | 55.20(3.6) | 2871(57.2%) | MVPA | right hip | 10 years |
| **cross-sectional studies** | | | | | | | |
| Araki et al., 2022 | JP | 791 elderly people from Company A | 83.1(5.9) | 139(64%) | SB;LPA;MVPA | thigh | － |
| Asai et al., 2018 | JP | the baseline data of a cohort study among elderly(≥60 y) | 71.5(7.0) | 1005(48.1%) | farming | wrist | － |
| Cruz et al., 2020 | USA | 3,233 US adults from the 2005-2006 NHANES | 47.43(19.45) | 3233(52.1%) | SB;LPA;MVPA | on the hip | － |
| Ba et al., 2024 | CN | The data from the most recent summary-level GWAS | n/r | 422218(n/r) | SB;PA;MVPA | upper thigh | － |
| Bae et al., 2023 | KR | 496 South Koreans older than 65 years old | 75.8 | 283(65.72%) | MVPA | nondominant wrist | － |
| Biddle et al., 2021 | AU | Project STAND、Walking Away from Diabetes and PROPELS | 59.1 | 1574(69.6%) | SB;MVPA | the thigh | － |
| Bustamante et al., 2013 | USA | Older Latino adults living in the Chicago, Illinois area | 65.97(9.12) | 174(73.6%) | SB;LPA;MVPA | buttock | － |
| Casals et al., 2024 | ES | 235 frail/frail elderly people living in a Spanish community | 74.42(7.77) | 235(66.8%) | LPA;MVPA;SB | nondominant wrist | － |
| Casanova et al., 2023 | UK | Participants from the UK Biobank | F:56.08(7.63);  M:57.19(7.75) | 145982(43.53%) | SB;PA | wrist | － |
| Chen et al., 2023 | TW | A medical center in Taipei City recruits community residents | 80.5(7.1) | 180(55.0%) | LPA;MVPA | hip | － |
| Choi et al., 2019 | USA | participants in the UK Biobank study | 40 to 69 years old | 377234(n/r) | n/r | the wrist | － |
| Dennison et al., 2021 | UK | UK Biobank | 56.2 | 95744(56.4%) | Moderate activity Step;SB; | wrist | － |
| Dhakal et al., 2023 | JP | Nakanojo Study | 75.17 (4.74) | 319 (50.2%) | PA | body | － |
| Eriksson et al., 2020 | SE | Healthy Ageing Initiative (HAI) | 70 | 3531(n/r) | SB | hip | － |
| Nobrega et al., 2023 | ES | 785 older adults from 10 European countries | 68.6(8.8) | 785(59.2%) | PA | thigh | － |
| Heinz et al., 2022 | USA | NHANES | 33.7(22.6) | 7162(51.7%) | PA;SB | right hip | － |
| Hollands et al., 2020 | UK | The participants who were taking part in the eMotion | 37.3(12.3) | 43(81.4%) | MVPA;LPA;LMVPA | wrist | － |
| Hsiao et al., 2022 | TW | Taiwanese community elders, aged above 65 | 80.2(7.0) | 167(49.1%) | SB;MVPA | the hip | － |
| Iob et al., 2023 | UK | the UK Biobank | n/r | MVPA:377,000; SB:91,000 | PA;SB | wrist | － |
| Kirschner et al, 2022 | NL | 374 participants at a Dutch vocational school | 18.8(2.8) | 85(70.6%) | PA;MVPA;LPA;SB | right thigh | － |
| Larisch et al., 2020 | SE | 662 participants from the same two Swedish companies | 41(9) | 662(68%) | LIPA;MVPA;SEB | left wrist | － |
| Lee et al., 2014 | USA | 810 adults age 60 and older | 70.37 | 810(54.85%) | PA | n/r | － |
| Li et al., 2022 | DE | 78 healthy adults | 25.46(6.18) | 78(73.08%) | PA | chests | － |
| Loprinzi et al., 2013 | USA | (NHANES)2005–2006 | 73.5 | 708(42.8%) | MVPA; LPA | on the right hip | － |
| Loprinzi et al., 2014 | USA | (NHANES) 2005–2006 | 46.3 | 2574(51.3%) | PA | n/r | － |
| Maher et al., 2018 | USA | Project MOBILE | 40.3(9.6) | 116(n/r) | MVPA | waist | － |
| McKercher et al., 2009 | AU | 1995 young adults in a 2004-2006 national stud | men:31.6 (2.6) ; women:31.4 (2.6) | 1995(52.4%) | PA | n/r | － |
| Michalak et al., 2022 | DE | 35 German MDD outpatients, 36 non-depressed controls | 39.57 (12.30) | 71(n/r) | PA | right leg | － |
| Morres et al., 2019 | GR | 19 outpatients from a community Mental Health Centre | 47.79(11.67） | 19(68.42%) | PA;MVPA;LPA;SB | right waist | － |
| Nobrega et al., 2023 | ES | SHARE | 68.6 | 785(59.2%) | PA | upper thigh | － |
| Park et al., 2024 | KR | 1543 participants from Republic of Korea | n/r | 1543(61.6%) | SB;LPA;MVPA | waist | － |
| Rethorst et al., 2017 | USA | HCHS/SOL | 41.06 | 16415(52.13%) | SB;LPA;MVPA | n/r | － |
| Song et al., 2012 | KR | 4,058 adults from NHANES | n/r | 4058(n/r) | SB;LPA;MVPA | right hip | － |
| Tully et al., 2020 | UK | 1360 participants from UK | 75.18 | 1360(62%) | SB;LPA;MVPA | dominant hip | － |
| Vallance et al., 2011 | CA | 2,862 adults from the 2005-2006 US NHANES | 45.7(13.7) | 2764(50.2 %) | SB;MVPA | right hip | － |
| Wemeck et al., 2022 | BR | 243 participants from southeast Brazil | 41.8(n/r) | 243(56.4%) | SB;LPA;MVPA | waistline | － |
| Yasunaga et al., 2018 | JP | 276 random Matsudo City, Japan residents | 74.4(5.3) | 276(38%) | SB;LPA;MVPA | waist | － |
| Zhou et al., 2024 | CN | 318 uni. students from three provinces | 21.13 | 318(60.1%) | SB;LPA;MVPA | right hipbone | － |
| FU:follow up.SD:standard deviation.n/a:not applicable.n/r:not reported.SB:sedentary behaviour.LPA:light physical activity.MVPA:moderate-to-vigorous physical activity.TPA:total physical activity.LIPA:light-intensity physical activity.LMVPA:light plus moderate-to-vigorous activity.METs:Metabolic Equivalents of Task.cpm:counts per minute.h/d:hours per day.min/day:minutes per day.mG:milli-G.Q=quartile.T=tertile.S=subgroup.IQR:Interquartile Range.Mdn:medianM:in male.F:in female.CA:Canada.SE:Sweden.UK:UnitedKingdom.AU:Australia.BR:Brazil.DE:Germany.TW:Taiwan.NL:Netherlands.PL:Poland.JP:Japan.CN:China.KR:Korea.ES:Spain.USA:United States of America.GR:Greece.uni.:university.LABS-2:Longitudinal Assessment of Bariatric Surgery-2.Birth Cohort 1936:Participants were drawn from the Lothian Birth Cohort 1936(LBC1936).Birth Cohort 1950:The West of Scotland Twenty-07 1950s.NHANES:7162 participants from the 2005-2006 National Health and Nutrition Examination Survey.NHANES:National Health and Nutrition Examination Survey 2005–2006.Project MOBILE:Measuring Our Behaviors in Living Environments.SHARE:the Survey of Health, Ageing and Retirement in Europe.HCHS/SOL:16,415 Hispanic/Latino adults.NHANES:National Health and Nutrition Examination Survey.JNAO:Japan's National Astronomical Observatory. | | | | | | | |

| **Table S.6 provides an overview of the included cohort and the relationship between sedentary behavior, physical activity, and depression** | | | | | |
| --- | --- | --- | --- | --- | --- |
| **Author(year)** | **SB behaviour** | | **Mean SB duration (h/day)** | **depression** **n (%)** | **Effect size OR** |
|  | **Reported as** | **Cut-off** |  |  |  |
| Araki et al., 2022 | SB | SB:<1.5METs | 9.38 h/d | 40(28.8%) | 1.083(0.98,1.15) |
| Ba et al., 2024 | SB | SB:>425milligravities | n/r | n/r | 1.02(1,1.05) |
| Biddle et al., 2021 | SB | SB:≥30min/day | 9.2 h/d | 40(28.8%) | 1.135(1.04,1.23) |
| Bustamante et al., 2013 | SB | SB:<100cpm | 9 h/d | 40(23%) |  |
| Casals et al., 2024 | SB | SB:≤ 57mG | n/r | 95(40.4%) | 0.99(0.981,1) |
| Casanova et al., 2023 | SB | n/r | 9.18 h/d | 34858(23.88%) | 0.96(0.86,1.07) |
| Cruz et al., 2020 | SB | SB:<100cpm | 9.82 h/d | n/r | 1.32(1.15,1.51) |
| Dennison et al., 2021 | SB | n/r | n/r | 6527(0.068%) |  |
| Eriksson et al., 2020 | SB | SB≤100cpm | n/r | 176（4.98%） | 1.031(1.007,1.055) |
| Hsiao et al., 2022 | SB | SB:<100cpm | 10.1±1.25 h/day | 26(15.6%) | 0.92(0.23,3.6) |
| Iob et al., 2023 | SB | SB:<100cpm | 10.1±1.25 h/day | 26(15.6%) |  |
| King et al., 2022 | SB | SB: 0 steps per minute | 6.6 (5.2-8.3) h/day | 190(29.50%) |  |
| Kirschner et al, 2022 | SB | SB:≤1.5METs | 8.3±1.6 h/day | 10(12.5%) | 3.1(0.877,10.84) |
| Konopka et al., 2022 | SB | SB:553.5-634.9min/d | 9.3±1.6 h/day | 203（9.8%) | 1.13(0.76,1.66) |
| Ku et al., 2017 | SB | SB:<100cpm | 8.72±3.08 h/day | n/r | 1.2(0.47,3.08) |
| Michalak et al., 2022 | SB | SB:<100cpm | n/r | n/r |  |
| Okely et al., 2019 | SB | n/r | LBC1936:62.51(SD=10.38); Twenty-07 1930s:68.16(10.93); Twenty-07 1950s:60.84(10.77) | n/r | 1.1503(1.02,1.29) |
| Park et al., 2024 | SB | SB:<100cpm | 6.79 h /day | n/r | 1.005(0.978,1.033) |
| Rahmani et al., 2023 | SB | SB:<100cpm | n/r | n/r |  |
| Rethorst et al., 2017 | SB | SB:<100 cts/min | 11.20 h /day | n/r | 1.12(1.04,1.21) |
| Siwa et al., 2023 | SB | SB:<100cpm | n/r | 500(63.7%) | 1.58(1.34,1.86) |
| Song et al., 2012 | SB | n/r | 13.79 h /day | 789(19.45%) |  |
| Tully et al., 2020 | SB | SB:<100cpm | n/r | n/r | 1.8(1.68,1.91) |
| Vallance et al., 2011 | SB | SB:<100cpm | 8.5±2.2 h/d | 195(6.813%) | 2.01(0.87,4.64) |
| Wemeck et al., 2022 | SB | SB:<100cpm | 8.1 h/day | 61(25.1%) | 1.04(0.97,1.11) |
| Yasunaga et al., 2018 | SB | SB:≤1.5METs | 8.74 h /day | 8(3%) | 1.14(1.02,1.26) |
| Zhou et al., 2024 | SB | SB:<100cpm | 9.95 h /day | 55(17.3%) | 1.37(1.09,3.63) |
| **Author(year)** | **LPA behaviour** | | **Mean LPA duration (min/day)** | **depression n (%)** | **Effect size:age** |
|  | **Reported as** | **Cut-off** |  |  |  |
| Araki et al., 2022 | LPA | LPA:1.6-2.9METs | 263.4 min/day | 40(28.8%) | 0.914(0.87,0.97) |
| Blodgett et al., 2023 | LPA | LPA:≥100 steps/min | 336 min/day | n/r | 0.66(0.47,0.93) |
| Bustamante et al., 2013 | LPA | LPA:100–1565cpm | 60 min/day | 40(23%) |  |
| Casals et al., 2024 | LPA | LPA:57-104 mG | 115.11min/day | 95(40.4%) | 0.997(0.992,1.003) |
| Chen et al., 2023 | LPA | LPA:100-2019cpm | 237.3 min/day | 33(18.3%) | 0.991(0.676,1.447) |
| Cruz, et al., 2020 | LPA | LPA:100-2020cpm | 304.85 min/day | n/r |  |
| Herbolsheimer et al., 2018 | LPA | LPA:5000–9999steps | n/r | n/r |  |
| Ho et al., 2022 | LPA | LPA:30-125mg(min/week) | 293±62 min/day | 1262(3.4%) | 0.94(0.63,1.38) |
| Hollands et al., 2020 | LPA | LPA:45.2-91.7mg | Mdn:7.1 (IQR:1.6-16.6) | 27(62.8%) | 1.0325(0.995,1.072) |
| Kirschner et al, 2022 | LPA | LPA:1.6–2.9METs | 294±90 min/day | 10(12.5%) | 0.24(0.059,0.99) |
| Ku et al., 2017 | LPA | LPA:100–1951cpm | 225±104.4 min/day | n/r | 0.66(0.48,0.91) |
| Larisch et al., 2020 | LIPA | LIPA:200–2689cpm | n/r | n/r | 2.03(0.42,10.01) |
| Li et al., 2022 | LPA | LPA:1.5-3METs | n/r | n/r | 0.98(0.97,0.99) |
| Loprinzi et al., 2013 | LPA | LPA:100-2019cpm | 294.5±4.9 min/day | 105(14.9%) | 0.8(0.67,0.95) |
| Michalak et al., 2022 | LPA | LPA:100-1534cpm | n/r | n/r |  |
| Park et al., 2024 | LPA | LPA:100-2019cpm | 496.7 min/day | n/r | 0.976(0.938,1.015) |
| Rahmani et al., 2023 | LPA | LPA:100-1534cpm | n/r | n/r |  |
| Rethorst et al., 2017 | LPA | LPA:100-1534cts/min | 217.53 min/day | n/r | 1.124(0.99,1.26) |
| Siwa et al., 2023 | LPA | LPA:100-2019cpm | n/r | 500(63.7%) |  |
| Song et al., 2012 | LPA | LPA:500-2019cpm | 130 min/day | 789(19.45%) |  |
| Tully et al., 2020 | LPA | LPA:100-2019cpm | n/r | n/r | 0.74(0.65,0.81) |
| Wemeck et al., 2022 | LPA | LPA:100-1,951cpm | n/r | 61(25.1%) |  |
| Yasunaga et al., 2018 | LPA | LPA:1.5-3.0METs | 328.8 min/day | 8(3%) | 0.87(0.77,0.99) |
| Zhou et al., 2024 | LPA | LPA:100–1952cpm; | 177.16 min/day | 55(17.3%) | 0.92(0.318,1.381) |
| **Author(year)** | **MVPA behaviour** | | **Mean MVPA duration (min/day)** | **depression n (%)** | **Effect size:age** |
|  | **Reported as** | **Cut-off** |  |  |  |
| Araki et al., 2022 | MVPA | MVPA:≥3.0METs | 8.7 min/day | 40(28.8%) | 1.33(0.82,2.14) |
| Ba et al., 2024 | MVPA | MVPA:>425milligravities | n/r | n/r | 0.96(0.91,1) |
| Biddle et al., 2021 | MVPA | MVPA:≥1952cpm | 150 min/day | 40(28.8%) | 0.99(0.9,1.1) |
| Blodgett et al., 2023 | MVPA | MVPA:≥100 steps/min | 46 min/day | n/r | 0.83(0.66,1.05) |
| Bustamante et al., 2013 | MVPA | MVPA:1566-6139cpm | 30 min/day | 40(23%) | 0.793(0.738,0.854) |
| Casals et al., 2024 | MVPA | MVPA:≥104mG | 32.94 min/day | 95(40.4%) | 0.99(0.981,1) |
| Chen et al., 2023 | MVPA | MVPA:≥ 2020cpm | 12.8 min/day | 33(18.3%) | 0.26(0.077,0.858) |
| Cruz et al., 2020 | MVPA | MVPA:≥2020cpm | 14.57 min/day | n/r |  |
| Dennison et al., 2021 | MVPA | n/r | n/r | 6,527(6.82%) | 0.183(0.05,0.67) |
| Herbolsheimer et al., 2018 | MVPA | MVPA:>9999steps/days | n/r | n/r |  |
| Ho et al., 2022 | MVPA | MVPA:>125400mg(min/week) | 69±33 min/week | 1262(3.4%) | 0.76(0.59,0.98) |
| Hollands et al., 2020 | MVPA | MVPA:91.7-134.4mg | n/r | 27(62.8%) | 1.046(0.974,1.122) |
| Hsiao et al., 2022 | MVPA | MVPA:≥150 min/week | 10.72±13.24 min/day | 26(15.6%) |  |
| King et al., 2022 | MVPA | MVPA:>80 steps per minute | 64.4 min/day | 190(29.50%) | 0.44(0.177,1.105) |
| Kirschner et al, 2022 | MVPA | MVPA:>3METs | 84±30 min/day | 10(12.5%) | 0.016(0.0002,1.422) |
| Konopka et al., 2022 | MVPA | MVPA:>78.5min/d | 19.2 min/d | 203（9.8%) | 0.5(0.47,0.54) |
| Ku et al., 2017 | MVPA | MVPA:>1951cpm | 9.6±37.2 min/day | n/r | 0.94(0.84,1.05) |
| Larisch et al., 2020 | MVPA | MVPA:≥2690cpm | 62 min/day | n/r | 1.197(0.453,3.133) |
| Loprinzi et al., 2013 | MVPA | MVPA:2020-5998cpm | 10.0±0.9 min/day | 105(14.9%) | 0.78(0.64,0.94) |
| Loprinzi et al.,2014 | MVPA | MVPA:2020-5998cpm | 24.1 min/day | 134(5%) | 0.46(0.22,0.93) |
| Maher et al., 2018 | MVPA | MVPA:>2020cpm | 22.06(16.19) min/day | n/r | 1.03(0.97,1.08) |
| Michalak et al., 2022 | MVPA | MVPA:1535-3961cpm | n/r | n/a |  |
| Park et al., 2024 | MVPA | MVPA:2020-5999cpm | 52.5 min/day | n/r | 0.817(0.678,0.985) |
| Rahmani et al.,2023 | MVPA | MVPA:> 1535cpm | 22.6 min/day | n/r | 1.19(0.86,1.65) |
| Rethorst et al., 2017 | MVPA | MPA:1535–3961cts/min | 19.51 min/day | n/r | 1.05(0.65,1.68) |
| Siwa et al., 2023 | MVPA | MPA:1952–5724cpm | n/r | 500(63.7%) |  |
| Song et al.,2012 | MVPA | MVPA:2020-5998cpm | 20.7 min/day | 789(19.45%) | 0.72(0.54,0.97) |
| Tully et al., 2020 | MVPA | MVPA:>2020cpm | n/r | n/r | 1.23(1.11,1.38) |
| Vallance et al., 2011 | MVPA | MVPA:≥1952cpm | 25.6±18.2 min/day | 195(6.813%) | 0.37(0.2,0.7) |
| Wemeck et al., 2022 | MVPA | MVPA:1,952-9,498cpm | 20.9 min/day | 61(25.1%) |  |
| Yasunaga et al., 2018 | MVPA | MVPA:≥3.0METs | 50.0 min/day | 8(3%) | 0.84(0.59,1.2) |
| Zhang et al., 2021 | MVPA | n/r | 22.69±28.9 min/day | 368 (15.6%) | 1.017(0.931,1.112) |
| Zhou et al., 2024 | MVPA | MVPA:>1952cpm | 49.74 min/day | 55(17.3%) | 0.87(0.23,0.93) |
| Asai et al., 2018 | farming | Short:≤7.0 h/week;Long:>7.0 h/week | n/r | 40(2.54%) |  |
| **Author(year)** | **TPA behaviour** | | **Mean PA duration (min/day)** | **depression n (%)** | **Effect size:age** |
|  | **Reported as** | **Cut-off** |  |  |  |
| Ba et al., 2024 | TPA | TPA>425milligravities | n/r | n/r | 0.95(0.91,0.99) |
| Casanova et al., 2023 | TPA | n/r | n/r | 34858(23.88%) | 0.93(0.88,0.97) |
| Dennison et al., 2021 | TPA | n/r | n/r | 6527(0.068%) | 0.819(0.819,0.905) |
| Dhakal et al., 2023 | TPA | TPA>3METs | n/r | n/r | 0.98(0.96,0.99) |
| Herbolsheimer et al., 2018 | TPA | n/r | n/r | n/r | 0.9(0.82,0.98) |
| Hollands et al., 2020 | TPA | LPA:45.2-91.7mg;MVPA:91.7-134.4mg;LMVPA:80.4-45.2mg | Mdn:10.1(IQR:2.8-23.2) | 27(62.8%) |  |
| Iob et al., 2023 | TPA | TPA≥150min/week | n/r | 26(15.6%) | 0.96(0.93,0.98) |
| Kirschner et al, 2022 | TPA | SB:<100cpm;LPA:100-1534cpm;MVPA:1535-3961cpm | 378±96 min/day | 10(12.5%) | 0.25(0.07,0.85) |
| Nobrega et al., 2023 | TPA | n/r | 28.1±19.8 min/day | n/r | 0.98(0.96,0.99) |
| **Author(year)** | **Daily steps behaviour** | | **Mean Daily steps duration (**steps/day**)** | **depression n (%)** | **Effect size:age** |
|  | **Reported as** | **Cut-off** |  |  |  |
| Chan et al., 2022 | Daily steps | 1000steps/day | n/r | 136(42.24)% | 0.81(0.73,0.89) |
| Chan et al., 2023 | Daily steps | 1000steps/day | n/r | 1332(1.84%) | 0.93(0.91,0.95) |
| Hsueh et al., 2020 | Daily steps | <3500steps/day;S2:3500~6999steps/day;≥7000steps/day | 4,733.91±3073.56 | 274(15.6%) | 0.7(0.53,0.93) |
| Hussenoeder et al., 2022 | Daily steps | SB:<5000steps/day;MVPA:5000–9999steps/day;HPA:>9999steps/day | 10 116.3±3660.0 | n/r | 0.84(0.74,0.95) |
| King et al., 2022 | Daily steps | SB: 0 steps per minute;MVPA: >80 steps per minute | 7183±5526-9396 | 190(29.50%) | 0.14(0.06,0.35) |
| Lee et al., 2014 | Daily steps | Sedentary:<5000 steps;low to moderate :5000–7499; high to very high:>7500 | n/r | n/r | 0.28(0.09,0.91) |
| McKercher et al., 2009 | Daily steps | <5000;5000–7499;7500–9999;10,000–12,499;12,500+ | n/r | men:56(5.9%);  women:123(11.8%) | 0.48(0.11,2.01) |
| A:accelerometer.P:pedometer.FU:follow up.SD:standard deviation.n/a:not applicable.n/r:not reported.SB:sedentary behaviour.LPA:light physical activity.MVPA:moderate-to-vigorous physical activity.  TPA:total physical activity.LIPA:light-intensity physical activity.LMVPA:light plus moderate-to-vigorous activity.VVPA:Very Vigorous PA.HPA:higher intensity physical activity.METs:Metabolic Equivalents of Task.  cpm:counts per minute.h/d:hours per day.min/day:minutes per day.mG:milli-G.Q=quartile.T=tertile.S=subgroup.IQR:Interquartile Range.Mdn:median.M:in male.F:infemale.  CA:Canada.SE:Sweden.UK:UnitedKingdom.AU:Australia.BR:Brazil.DE:Germany.TW:Taiwan.NL:Netherlands.PL:Poland.JP:Japan.CN:China.KR:Korea.ES:Spain.  USA:United States of America.GR:Greece.uni.:university.LABS-2:Longitudinal Assessment of Bariatric Surgery-2.  Birth Cohort 1936:Participants were drawn from the Lothian Birth Cohort 1936(LBC1936).Birth Cohort 1950:The West of Scotland Twenty-07 1950s.NHANES:7162 participants from the 2005-2006 National Health and Nutrition Examination Survey.NHANES:National Health and Nutrition Examination Survey 2005–2006.Project MOBILE:Measuring Our Behaviors in Living Environments.SHARE:the Survey of Health, Ageing and Retirement in Europe.  HCHS/SOL:16,415 Hispanic/Latino adults.NHANES:National Health and Nutrition Examination Survey.JNAO:Japan's National Astronomical Observatory. | | | | | |

| **Table S.7 Characteristics of measurement methods used to assess instrumented sedentary behaviour and physical activity.** | | | | | | | |
| --- | --- | --- | --- | --- | --- | --- | --- |
| **Author** | **A/P** | **Device** | **Physical activity measures** | | **Assessment of valid days** | | **Mean wearing duration  (min/day)** |
|  |  |  | **Reported as** | **Cut-off value** | **Hours/day** | **Valid days** |  |
| **cohort studies** | | | | | | | |
| Blodgett et al., 2023 | A | activPAL3 micro | MVPA;LPA;SB | ≥100 steps/min | ≥10 hours perday for≥4 days | 7 | 15-16 h/d |
| Chan et al., 2022 | A | McRoberts | Daily steps | 1000 steps a day | n/r | 7 | n/r |
| Chan et al., 2023 | A | AX3 by Axivity Ltd | Daily steps | 1000 steps a day | n/r | 7 | n/r |
| Herbolsheimer et al., 2018 | A | activPAL™ | TPA | n/r | n/r | 7 | 24 h/d |
| Ho et al., 2022 | A | Axivity AX3 | MVPA;LPA;VPA | LPA:30-125mg;MPA:125-400mg;VPA:>400mg(min/week) | ≥10 hours perday for≥6 days | 7 | 6.91 h/d |
| Hsueh et al., 2020 | A | ActiGraph GT3X+ | Daily steps | <3500 steps/day;S2:3500~6999steps/day; ≥7 000 steps/day | ≥10 hours perday for≥5 days | 7 | 14.95 h/d |
| Hsueh et al., 2021 | A | Acti Graph GT3X + | Daily steps | <3500 steps/day; 3500~6999 steps/day; ≥7 000 steps/day | ≥10 hours perday for≥5 days | 7 | 14.95 h/d |
| King et al., 2022 | P | StepWatch™ | MVPA;SB | SB: 0 steps per minute;MVPA:>80 steps per minute | n/r | 7 | n/r |
| Konopka et al., 2022 | A | ActivPAL3™ | HPA;LPA;SB | SB:553.5-634.9 min/d;LPA :78.5-111.7 min/d. HPA:9.1-22.4 min/d | ≥10 h | 8 | 15 h/day |
| Ku et al., 2017 | A | GT3X+,ActiGraph | SB;LPA;MVPA | SB:<100cpm; LPA:100–1951cpm; MVPA:>1951cpm | ≥10 hours perday for≥5 days | 7 | n/r |
| Okely et al., 2019 | A | activPAL | SB | n/r | 24 hours a day for 7 days | 7 | 24 h/day |
| Rahmani et al.,2023 | A | Actical accelerometer | SB;LPA;MVPA | SB:< 100cpm;LPA:100-1534cpm;MVPA:>1535cpm | ≥10 hours perday for≥3 days | 7 | n/r |
| Siwa et al., 2023 | A | ActiGraph GT3X-BT | SB;LPA;MVPA | SB:<100cpm;LPA:100-2019cpm;MVPA:≥2020cpm | ≥8 hours perday for≥3days | 3 | 13.40 h/d |
| cross-sectional studies | | | | | | | |
| Araki et al., 2022 | A | ActivestyleProHJA-75C | SB;LPA;MVPA | SB:<1.5METs;LPA:1.6-2.9METs;MVPA:≥3.0 METs | ≥10 hours perday for≥5 days | 7 | n/r |
| Asai et al., 2018 | A | Actiwatch 2 | farming | Short:≤7.0 h/week;Long:>7.0 h/week | Daylength (sunrise to sunset) from JNAO | 2 | n/r |
| Cruz et al., 2020 | A | ActiGraph AM-7164 | LIPA;MVPA;SB | SB:<100cpm;LIPA:100-2020cpm;MVPA:≥2020cpm | 24 hours per day | 7 | n/r |
| Ba et al., 2024 | A | n/r | SB;TPA;MVPA | > 425 milligravities | n/r | n/r | n/r |
| Biddle et al., 2021 | A | ActiGraph LLC | SB;MVPA | SB:≥30min/day;MVPA:≥1952cpm | 24 hours perday for 7 days | 10 | 15.7 h/d |
| Bustamante et al., 2013 | A | GT3X ActiGraph | SB;LPA;MVPA | SB:<100cpm;LPA:100–1565cpm;MVPA:1566-6139cpm; | ≥10 hours perday for≥5 days | 7 | 15.7 h/d |
| Casals et al., 2024 | A | GENEActiv | LPA;MVPA;SB | SB:≤ 57mG;LPA:57-104mG;MPA:≥104-245 mG;VPA≥ 245 mG | ≥16 hours perday for≥4 days | 6 | 16 h/d |
| Casanova et al., 2023 | A | n/r | SB;PA | n/r | n/r | 7 | n/r |
| Chen et al., 2023 | A | ActiGraph GT3X+ | LPA;MVPA | LPA:100-2019cpm;MVPA:≥ 2020cpm | ≥10 hours perday for≥4 days | 7 | 10 h/d |
| Choi et al., 2019 | A | n/r | n/r | n/r | n/r | 7 | n/r |
| Dennison et al., 2021 | A | Axivity AX3 tri-axial | TPA | n/r | n/r | 7 | n/r |
| Dhakal et al., 2023 | A | n/r | TPA | > 3 METs | n/r | n/r | n/r |
| Eriksson et al., 2020 | A | ActiGraph GT3X+ | PA;SB | SB≤100cpm;MVPA:≥150 min/week;VPA:≥75min/week | n/r | 7 | n/r |
| Nobrega et al., 2023 | A | Axivity AX3 | PA | 28.1 (19.8) | 24 hours per day | 8 | n/r |
| Hollands et al., 2020 | A | GENEActiv | MVPA;LPA;  LMVPA | LPA:45.2-91.7mg;MVPA:91.7-134.4mg;LMVPA:80.4-45.2mg | ≥10 hours perday for≥4 days | 7 | 10 h/day |
| Hsiao et al., 2022 | A | ActiGraph wGT3X-BT | SB;MVPA | SB:<100cpm | ≥10 hours perday for≥4 days | 7 | 10 h/day |
| Hsueh et al., 2020 | A | ActiGraph GT3X+ | Daily step | <3500;3500–6999;≥ 7000 | ≥10 h per day | 7 | n/r |
| Hussenoeder et al.,2022 | P | SenseWear Pro3 devices | Daily step | SB:<5000steps/day;MVPA:5000–9999steps/day;HPA:>9999steps/day | n/r | n/r | n/r |
| Iob et al., 2023 | A | accelerometer | PA;SB | MVPA:≥150min/week | n/r | 7 | n/r |
| Kirschner et al., 2022 | A | ActivPAL3TM | PA;MVPA;LPA;SB | MVPA:>3METs;LPA:1.6–2.9METs; SB:≤1.5METs | ≥24 hours perday for≥4days | 4 | 24 h/day |
| Ku et al., 2017 | A | ActiGraph GT3X+ | SB;LPA;MVPA | SB:<100cpm;LPA:100–1951cpm;MVPA:>1951cpm | ≥10 h per day | 7 | n/r |
| Larisch et al., 2020 | A | ActiGraphTM GT3X | SB;LIPA;MVPA | MVPA:≥2690cpm;LPA:200–2689cpm;SB:1–200cpm | ≥2.5 hours perday for≥4days | 4 | n/r |
| Lee et al., 2014 | A | Actigraph AM-7164 | Daily step | n/r | ≥10 h per day | 7 | n/r |
| Li et al., 2022 |  | n/r | SB;LPA;MPA | SB:≤1.5METs;LPA:1.5-3METs;MPA:3-6 METs;VPA:≥6METs | n/r | n/r | n/r |
| Loprinzi et al., 2013 | A | ActiGraph 7164 | LPA;MVPA | LPA:100-2019cpm;MVPA:2020-5998cpm | ≥10 hours perday for≥4 days | 7 | n/r |
| Loprinzi et al., 2014 |  | ActiGraph 7164 | MVPA | MVPA:>2020cpm | at least 4 days | 7 | n/r |
| Maher et al., 2018 | A | Actigraph GT2M | MVPA | MVPA:>2020cpm | ≥10 hours perday | n/r | n/r |
| McKercher et al., 2009 | P | Yamax Digiwalker SW-200 | Daily step | <5000;5000–7499;7500–9999;10,000–12,499;12,500+ | >8 hours perday for≥4 days | 7 | n/r |
| Michalak et al., 2022 | A | Vitamove | SB;LPA;MVPA | SB:<100cpm;LPA:100-1534cpm;MVPA:1535-3961cpm | ≥10 hours perday for≥2 days | 2 | 14 h/day |
| Morres et al., 2019 | A | Actigraph GT3X+ | LPA;MVPA;TPA;SB;Step | SB:<100 cnts min^-1^;LPA: 100-1951 cnts min ^- 1^;  MVPA: 1952-9498 cnts min^- 1^;VVPA:≥9499 cnts min^- 1^; | >8 hours perday for≥3 days | 7 | 8 h/day |
| Nobrega et al.,2023 |  | Axivity AX3 | TPA | n/r | ≥16 h | ≥4 | 8 h/day |
| Park et al., 2024 | A | ActiGraph GT3X+ | SB;LPA;MVPA | SB:<100cpm;LPA:100-2019cpm;MVPA:2020-5999cpm | n/r | 7 | n/r |
| Rethorst et al., 2017 | A | Actical B-1 version | SB;LPA;MVPA | SB:<100 cts/min;LPA:100-1534 cts/min;MPA:1535–3961cts/min | ≥10 hours perday | 3 | 20 h/day |
| Song et al., 2012 | A | ActiGraph® AM-7164 | SB;LPA;MVPA | SB:n/r;LPA:500-2019counts/min;MVPA:2020-5998 counts/min | ≥10 hours perday for≥1day | 1 | 10 h/day |
| Tully et al., 2020 | A | ActiGraph wGT3X-BT+ | SB;LPA;MVPA | SB:<100cpm;LPA:100-2019cpm;MVPA:>2020cpm | ≥10 hours perday for≥4days | 4 | n/r |
| Vallance et al., 2011 | A | ActiGraph AM-7164 | SB; MVPA | MVPA:≥1952cpm;LPA:100–1951cpm;SB:<100cpm | ≥10 hours perday for≥4days | 7 | 14.6 h/day |
| Wemeck et al., 2022 | A | Actigraph GT3X | SB;LPA;MVPA | SB:100cpm;LPA:100-1951cpm;MVPA:1952-9498cpm | 4 hours/day for≥5days | 5 | 13.5 h/day |
| Yasunaga et al., 2018 | A | Active style ProHJA-350IT | SB;LPA;MVPA | SB:≤1.5METs;LPA:1.5-3.0 METs;MVPA:≥3.0 METs | 15 hours/day for≥7days | 7 | 15 h/day |
| Zhang et al.， 2021 | A | ActiGraph 7164 | MVPA | 22.69 ± 28.9min/day | 24 hours per day | 7 | n/r |
| Zhou et al., 2024 | A | ActiGraph wGT3X-BT | SB;LPA;MVPA | SB:< 100cpm;LPA:100–1952cpm;MVPA:>1952cpm | ≥10 hours perday for≥4days | 7 | 13.73 h/day |
| A:accelerometer.P:pedometer.FU:follow up.SD:standard deviation.n/a:not applicable.n/r:not reported.SB:sedentary behaviour.LPA:light physical activity.MVPA:moderate-to-vigorous physical activity.  TPA:total physical activity.LIPA:light-intensity physical activity.LMVPA:light plus moderate-to-vigorous activity.VVPA:Very Vigorous PA.HPA:higher intensity physical activity.METs:Metabolic Equivalents of Task.  cpm:counts per minute.h/d:hours per day.min/day:minutes per day.mG:milli-G.Q=quartile.T=tertile.S=subgroup.IQR:Interquartile Range.Mdn:median.M:in male.F:infemale.  CA:Canada.SE:Sweden.UK:UnitedKingdom.AU:Australia.BR:Brazil.DE:Germany.TW:Taiwan.NL:Netherlands.PL:Poland.JP:Japan.CN:China.KR:Korea.ES:Spain.  USA:United States of America.GR:Greece.uni.:university.LABS-2:Longitudinal Assessment of Bariatric Surgery-2.  Birth Cohort 1936:Participants were drawn from the Lothian Birth Cohort 1936(LBC1936).Birth Cohort 1950:The West of Scotland Twenty-07 1950s.NHANES:7162 participants from the 2005-2006 National Health and Nutrition Examination Survey.NHANES:National Health and Nutrition Examination Survey 2005–2006.Project MOBILE:Measuring Our Behaviors in Living Environments.SHARE:the Survey of Health, Ageing and Retirement in Europe.  HCHS/SOL:16,415 Hispanic/Latino adults.NHANES:National Health and Nutrition Examination Survey.JNAO:Japan's National Astronomical Observatory. | | | | | | | |

| **Table S.8 Characteristics of included cohorts** | | | | | | | | | | | | | | | | | | | | | | |
| --- | --- | --- | --- | --- | --- | --- | --- | --- | --- | --- | --- | --- | --- | --- | --- | --- | --- | --- | --- | --- | --- | --- |
| cross-sectional studies | Selection | | | | | | | | | | | | | Comparability | | Outcome | | | | | | Quality score（10） |
| Author (year) | Representativeness of the sample（*） | | | | Sample size（*） | | | Non-respondents（*） | | | Ascertainment of the exposure (risk factor)（**） | | | Comparability and control of confounding factors（**） | | Assessment of outcome（**） | | | | Statistical test（*） | |  |
|  | a | b | c | d | a | b | c | a | b | c | a | b | c | a | b | a | b | c | d | a | b |  |
| Asai et al., 2018 | * |  |  |  | * |  |  | * |  |  | ** |  |  | ** |  |  |  | * |  | * |  | 9 |
| Araki et al., 2022 | * |  |  |  | * |  |  | * |  |  | ** |  |  | ** |  |  |  | * |  | * |  | 9 |
| Ba et al., 2024 | * |  |  |  | * |  |  | * |  |  | ** |  |  | ** |  |  |  | * |  | * |  | 9 |
| Biddle et al., 2021 | * |  |  |  | * |  |  | * |  |  | ** |  |  | ** |  |  |  | * |  | * |  | 9 |
| Bustamante et al., 2013 | * |  |  |  | * |  |  | * |  |  | ** |  |  | ** |  |  |  | * |  | * |  | 9 |
| Casals et al., 2024 | * |  |  |  | * |  |  | * |  |  | ** |  |  | * |  |  |  | * |  | * |  | 8 |
| Chen et al., 2023 | * |  |  |  | * |  |  | * |  |  | ** |  |  | ** |  |  |  | * |  | * |  | 9 |
| Casanova et al., 2023 | * |  |  |  | * |  |  | * |  |  | ** |  |  | ** |  |  |  | * |  | * |  | 9 |
| Choi et al., 2019 | * |  |  |  | * |  |  | * |  |  | ** |  |  | － |  |  |  | * |  | * |  | 7 |
| Cruz, et al.2020 | * |  |  |  | * |  |  | * |  |  | ** |  |  | ** |  |  |  | * |  | * |  | 9 |
| Dennison et al., 2021 | * |  |  |  | * |  |  | * |  |  | ** |  |  | * |  |  |  | * |  | * |  | 8 |
| Dhakal et al., 2023 | * |  |  |  | * |  |  | * |  |  | ** |  |  | ** |  |  |  | * |  | * |  | 9 |
| Eriksson et al., 2020 | * |  |  |  | * |  |  | * |  |  |  | * |  | ** |  |  |  | * |  | * |  | 8 |
| Hollands et al., 2020 |  | * |  |  | * |  |  | * |  |  | ** |  |  | ** |  |  |  | * |  | * |  | 9 |
| Hsiao et al., 2022 |  | * |  |  | * |  |  | * |  |  | ** |  |  | * |  |  |  | * |  | * |  | 8 |
| Iob et al., 2023 | * |  |  |  | * |  |  | * |  |  | ** |  |  | ** |  |  |  | * |  | * |  | 9 |
| Kirschner et al, 2022 |  | * |  |  | * |  |  | * |  |  | ** |  |  | ** |  |  |  | * |  | * |  | 9 |
| Larisch et al., 2020 |  | * |  |  | * |  |  | * |  |  | ** |  |  | － |  |  |  | * |  | * |  | 5 |
| Lee et al., 2014 | * |  |  |  | * |  |  | * |  |  | ** |  |  | ** |  |  |  | * |  | * |  | 9 |
| Li et al., 2022 | * |  |  |  | － |  |  | － |  |  | ** |  |  | － |  |  |  | * |  | * |  | 7 |
| Loprinzi et al., 2013 | * |  |  |  | * |  |  | * |  |  | ** |  |  | － |  |  |  | * |  | * |  | 7 |
| Loprinzi et al.,2014 | * |  |  |  | * |  |  | * |  |  | ** |  |  | ** |  |  |  | * |  | * |  | 9 |
| Maher et al., 2018 | * |  |  |  | * |  |  | * |  |  | ** |  |  | ** |  |  |  | * |  | * |  | 9 |
| McKercher et al., 2009 | * |  |  |  | * |  |  | * |  |  |  | * |  | ** |  |  |  | * |  | * |  | 8 |
| Morres et al., 2019 |  | * |  |  | * |  |  | * |  |  | ** |  |  | － |  |  |  | * |  | * |  | 6 |
| Michalak et al., 2022 |  | * |  |  | － |  |  | * |  |  | ** |  |  | ** |  |  |  | * |  | * |  | 9 |
| Nobrega et al., 2023 |  | * |  |  | * |  |  | * |  |  | ** |  |  | ** |  |  |  | * |  | * |  | 9 |
| Park et al., 2024 |  | * |  |  | * |  |  | * |  |  | ** |  |  | ** |  |  |  | * |  | * |  | 9 |
| Rethorst et al., 2017 | * |  |  |  | * |  |  | * |  |  | ** |  |  | ** |  |  |  | * |  | * |  | 8 |
| Song et al.,2012 | * |  |  |  | * |  |  | － |  |  | ** |  |  | ** |  |  |  | * |  | * |  | 8 |
| Tully et al., 2020 |  | * |  |  | * |  |  | － |  |  | ** |  |  | ** |  |  |  | * |  | * |  | 9 |
| Vallance et al., 2011 | * |  |  |  | * |  |  | * |  |  | ** |  |  | ** |  |  |  | * |  | * |  | 8 |
| Wemeck et al., 2022 | * |  |  |  | * |  |  | － |  |  | ** |  |  | ** |  |  |  | * |  | * |  | 8 |
| Yasunaga et al., 2018 | * |  |  |  | * |  |  | － |  |  | ** |  |  | ** |  |  |  | * |  | * |  | 8 |
| Zhou et al., 2024 | * |  |  |  | * |  |  | － |  |  | ** |  |  | ** |  |  |  | * |  | * |  | 9 |
| cohort study | Selection | | | | | | | | | | | | | Comparability | | Outcome | | | | | | Quality score（9） |
| Author (year) | Representativeness of the exposed cohort (adult mixed gender or male or female)（*） | | | | Selection of the non-exposed cohort（*） | | | Ascertainment of exposure（*） | | | Demonstration that outcome of interest was not present at start of study（*） | | | Comparability of cohorts on the basis of the design or analysis（**） | | Assessment of outcome（*） | | Was follow-up long enough for outcomes to occur（*） | | Adequacy of follow up of（*） | |  |
| Blodgett et al., 2023 | * | | | | * | | | * | | | * | | | ** | | * | | * | | * | | 9 |
| Chan et al., 2022 | * | | | | * | | | * | | | * | | | ** | | * | | * | | － | | 8 |
| Chan et al., 2023 | * | | | | * | | | * | | | * | | | ** | | * | | * | | － | | 8 |
| Ho et al., 2022 | * | | | | * | | | * | | | * | | | ** | | * | | * | | * | | 9 |
| Herbolsheimer et al., 2018 | * | | | | * | | | * | | | * | | | － | | * | | * | | * | | 7 |
| King et al., 2022 | * | | | | * | | | * | | | * | | | ** | | * | | * | | * | | 9 |
| Konopka et al., 2022 | * | | | | * | | | * | | | * | | | ** | | * | | * | | * | | 9 |
| Okely et al., 2019 | * | | | | * | | | * | | | * | | | ** | | * | | * | | * | | 9 |
| Rahmani et al.,2023 | * | | | | * | | | * | | | * | | | * | | * | | * | | － | | 7 |
| Siwa et al., 2023 | * | | | | * | | | * | | | * | | | ** | | * | | － | | * | | 8 |
| Ku et al.,2017 | * | | | | * | | | * | | | * | | | ** | | * | | * | | * | | 9 |
| Hsueh et al.，2020 | * | | | | * | | | * | | | * | | | * | | * | | * | | － | | 7 |
| Hussenoeder et al., 2022 | * | | | | * | | | * | | | * | | | * | | * | | * | | － | | 7 |
| Zhang et al., 2021 | * | | | | * | | | * | | | * | | | * | | * | | * | | * | | 8 |

**Supplementary Material Fig. SA-G.**


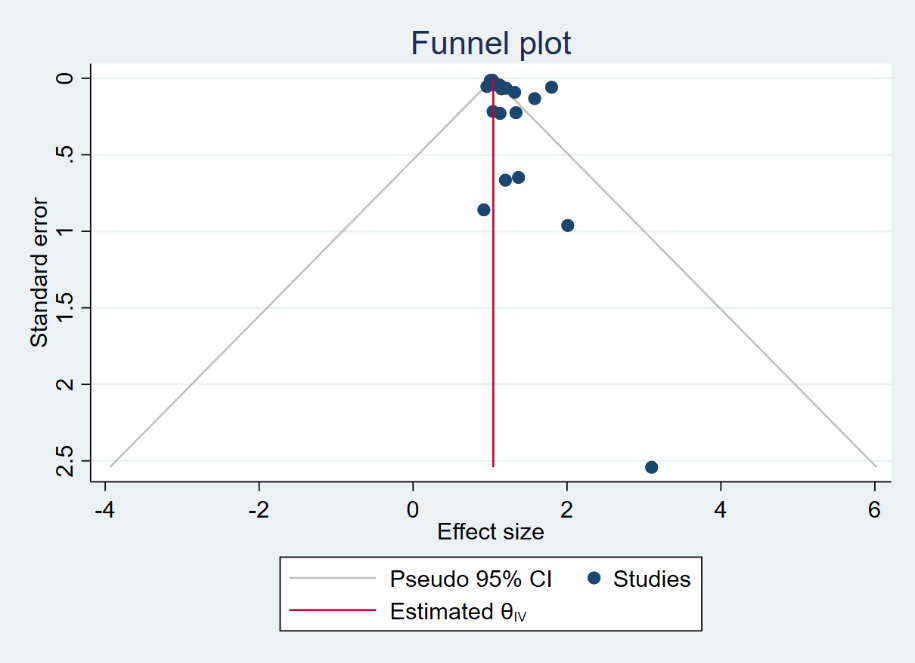


Fig. SA Funnel plot for publication bias


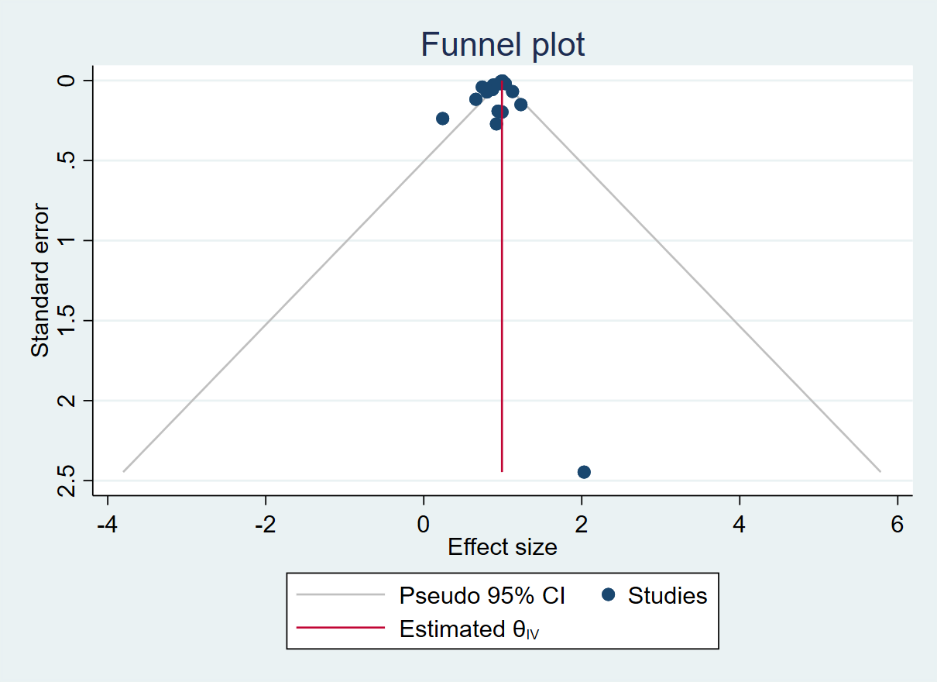


Fig. SB Funnel plot for publication bias.


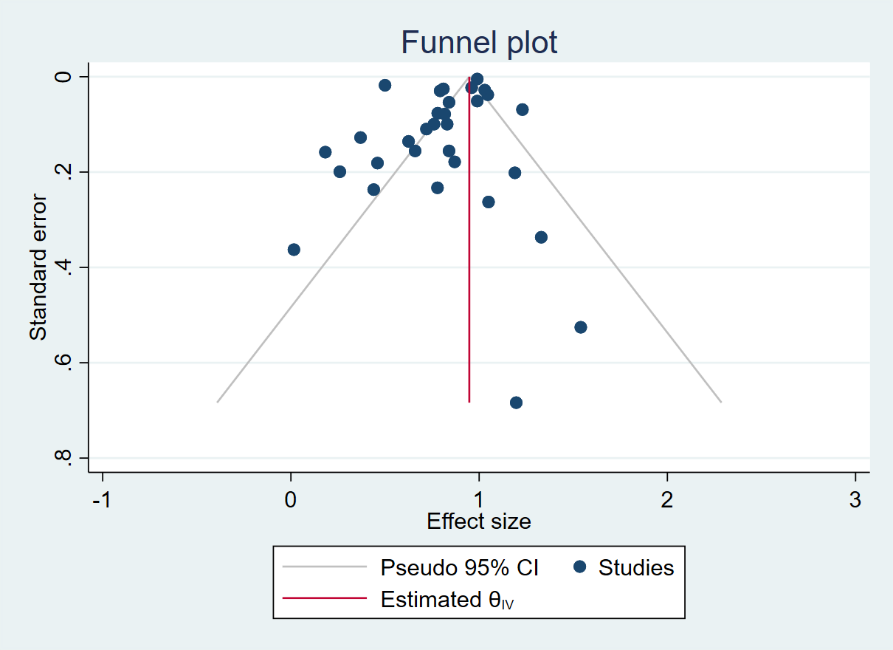


Fig. SC Funnel plot for publication bias.


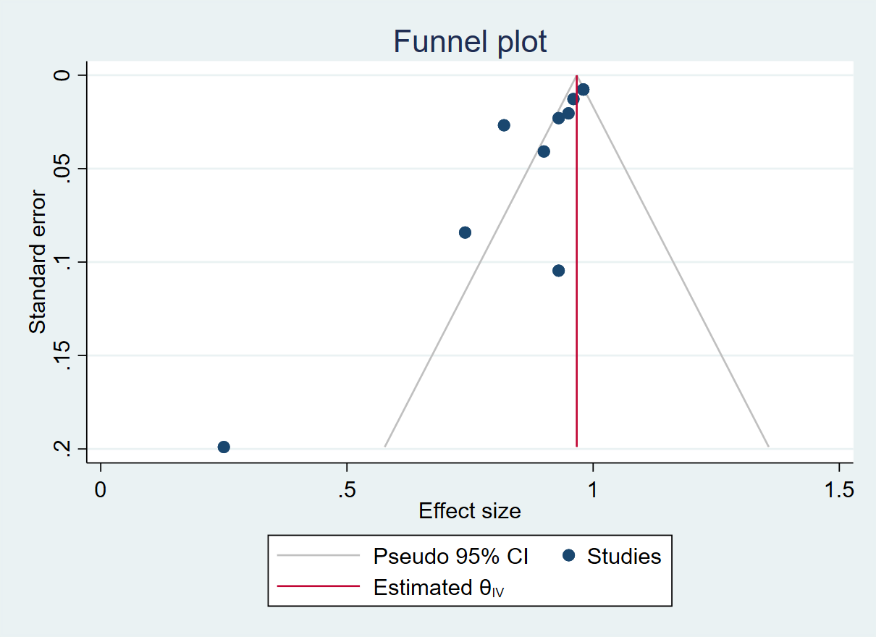


Fig. SD Funnel plot for publication bias.


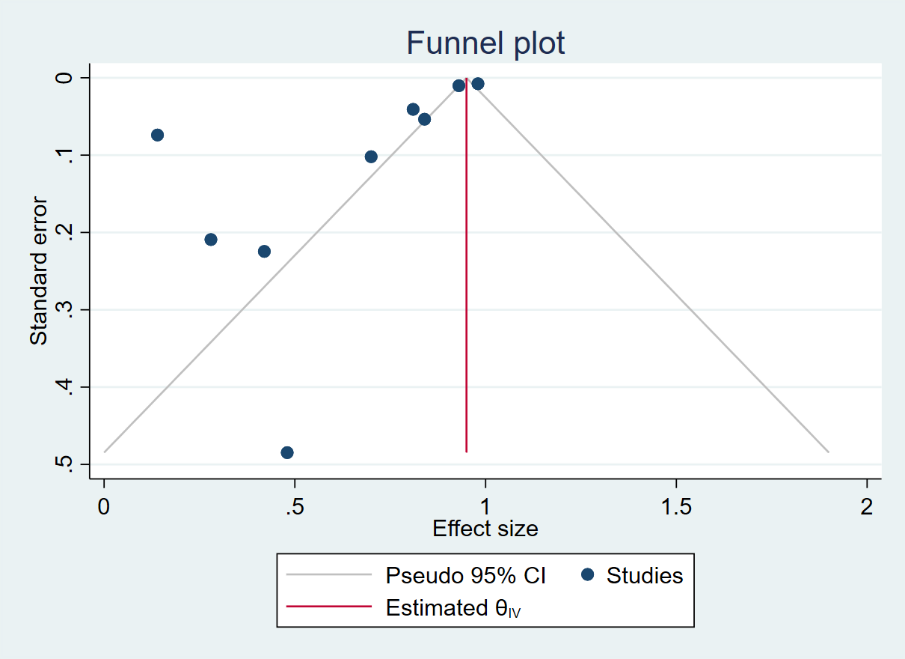


Fig. SE Funnel plot for publication bias.


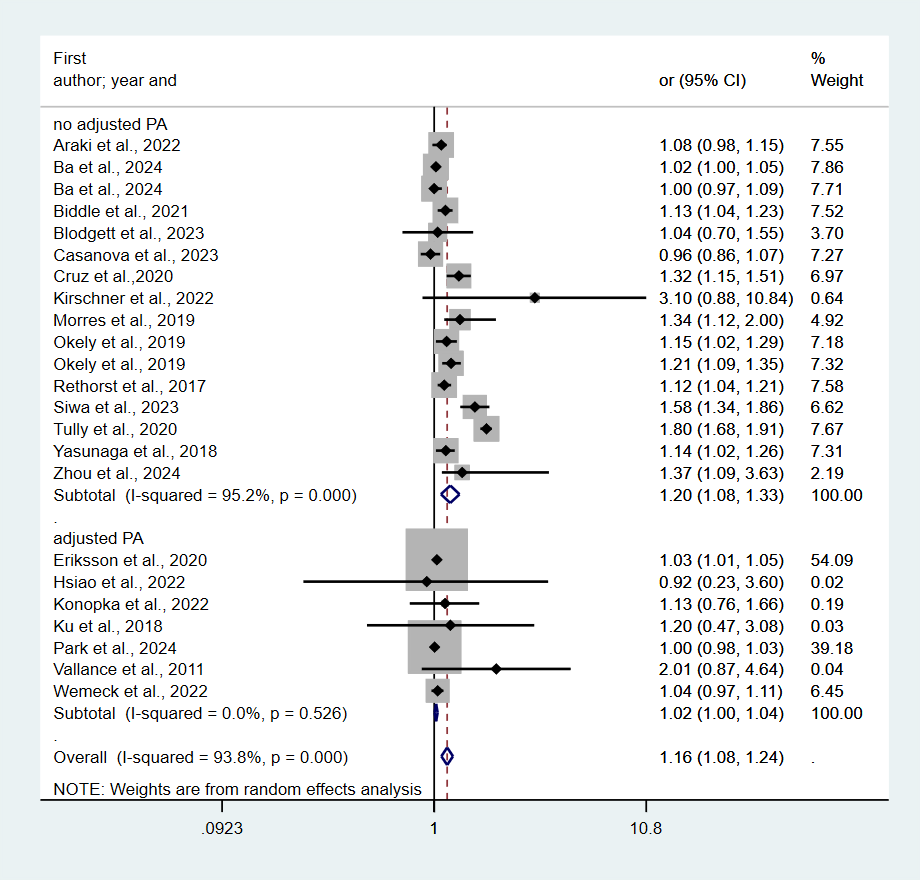


Fig. SH Forest plots of the association between instrumented SB and depression after PA adjustment


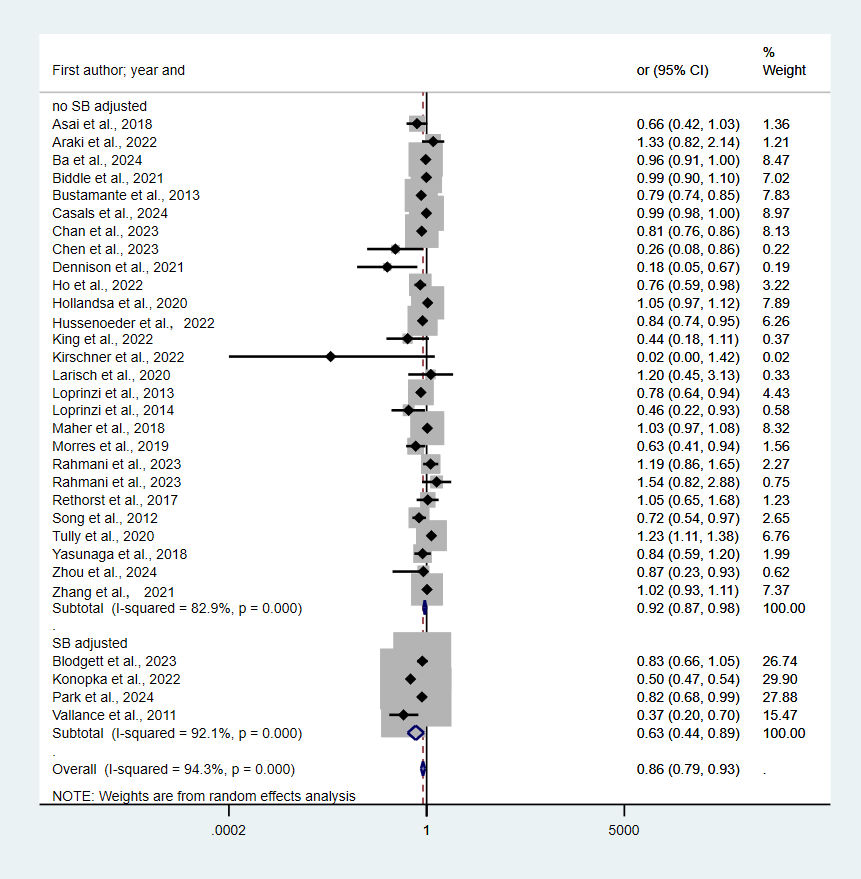


Fig. SG Forest plots of the association between instrumented SB and depression after PA adjustment
